# *RB1*-Negative Retinal Organoids Display Proliferation of Cone Photoreceptors and Loss of Retinal Differentiation

**DOI:** 10.3390/cancers14092166

**Published:** 2022-04-26

**Authors:** Deniz Kanber, Julia Woestefeld, Hannah Döpper, Morgane Bozet, Alexandra Brenzel, Janine Altmüller, Fabian Kilpert, Dietmar Lohmann, Claudia Pommerenke, Laura Steenpass

**Affiliations:** 1 Institute of Human Genetics, University Hospital Essen, University of Duisburg-Essen, 45147 Essen, Germany; deniz.kanber@uni-due.de (D.K.); julia.woestefeld@uni-due.de (J.W.); hannah.doepper@uk-essen.de (H.D.); morgane.bozet@stud.thga.de (M.B.); fabian.kilpert@uni-due.de (F.K.); dietmar.lohmann@uni-due.de (D.L.); 2 Eye Oncogenetics Research Group, University Hospital Essen, University of Duisburg-Essen, 45147 Essen, Germany; 3 Imaging Center Essen (LMU), Institute for Experimental Immunology and Imaging, University Hospital Essen, University of Duisburg-Essen, 45147 Essen, Germany; alexandra.brenzel@uk-essen.de; 4 West German Genome Center (WGGC), University of Cologne, 50931 Cologne, Germany; janine.altmueller@bih-charite.de; 5 Core Facility Genomics, Berlin Institute of Health at Charite, Universitätsmedizin Berlin, 10115 Berlin, Germany; 6 Max Delbrück Center for Molecular Medicine in the Helmholtz Association, 13125 Berlin, Germany; 7 Bioinformatics and Databases, Leibniz Institute DSMZ—German Collection of Microorganisms and Cell Cultures, 38124 Braunschweig, Germany; claudia.pommerenke@dsmz.de; 8 Human and Animal Cell Lines, Leibniz Institute DSMZ—German Collection of Microorganisms and Cell Cultures, 38124 Braunschweig, Germany

**Keywords:** retinoblastoma, stem cells, retinal organoids, RNA-seq

## Abstract

**Simple Summary:**

Retinoblastoma is a tumor of the eye’s retina, which is the very specialized tissue responsible for vision. In 98% of cases, the tumor is caused by inactivation of the *RB1* gene. Due to lack of material and models, the understanding of *RB1* mutations in tumor development is still unsatisfactory. We aimed to establish a human laboratory model for retinoblastoma. While differentiating stem cells with a mutation in *RB1* into retina, we observed reduced differentiation potential but enhanced proliferation—general hallmarks of tumor development. The gene expression signature in the model resembled that of tumor material. This approach now enables research on retinoblastoma and probably therapy in the correct tissue, the human retina.

**Abstract:**

Retinoblastoma is a tumor of the eye in children under the age of five caused by biallelic inactivation of the *RB1* tumor suppressor gene in maturing retinal cells. Cancer models are essential for understanding tumor development and in preclinical research. Because of the complex organization of the human retina, such models were challenging to develop for retinoblastoma. Here, we present an organoid model based on differentiation of human embryonic stem cells into neural retina after inactivation of *RB1* by CRISPR/Cas9 mutagenesis. Wildtype and *RB1* heterozygous mutant retinal organoids were indistinguishable with respect to morphology, temporal development of retinal cell types and global mRNA expression. However, loss of pRB resulted in spatially disorganized organoids and aberrant differentiation, indicated by disintegration of organoids beyond day 130 of differentiation and depletion of most retinal cell types. Only cone photoreceptors were abundant and continued to proliferate, supporting these as candidate cells-of-origin for retinoblastoma. Transcriptome analysis of *RB1* knockout organoids and primary retinoblastoma revealed gain of a retinoblastoma expression signature in the organoids, characterized by upregulation of *RBL1* (p107), *MDM2*, *DEK*, *SYK* and *HELLS*. In addition, genes related to immune response and extracellular matrix were specifically upregulated in *RB1*-negative organoids. In vitro retinal organoids therefore display some features associated with retinoblastoma and, so far, represent the only valid human cancer model for the development of this disease.

## 1. Introduction

Retinoblastoma is a rare tumor of the neural retina in children mainly diagnosed before the age of five. The main cause of retinoblastoma is biallelic inactivation of *RB1*, which can occur as part of a heritable tumor predisposition disease. So far, *BCOR* is the only gene recurrently mutated in retinoblastoma, found in about 10% of cases, leaving inactivation of *RB1* as the main driver of tumor formation [1,2,3]. About 1–2% of patients with retinoblastoma show no *RB1* alteration but instead amplification of *MYCN* [4]. Retinoblastoma progression appears to be associated with a shift in the transcriptome pattern from photoreceptor to RNA-metabolism associated genes [5,6,7,8]. Models to understand retinoblastoma development and the role of *RB1* therein have been established in mice, human fetal retina tissue, cell lines and most recently as 3D retinal organoids. Cell lines are of value in pharmacological studies investigating target gene chemosensitivity and -resistance to certain cytotoxic drugs and often serve as controls in xenograft models [9,10]. However, they represent the end stage of retinoblastoma development and are unsuitable for studies on tumor initiation and development. Studies in mice cannot be translated to humans because the two species exhibit non-homologous retinoblastoma development. Thus, homozygous knockout of *Rb1* in mice is lethal in the embryo and heterozygous animals frequently develop pituitary tumors, but not retinoblastoma [11]. Models using conditional knockout of *Rb1* in the retina on *p107*^−/−^ and/or *p130*^−/−^ backgrounds result in loss of layered retina structure and impaired retinal differentiation [11,12,13,14]. Histopathological studies on mouse retinoblastoma pointed to an amacrine/horizontal cell-of-origin rather than a photoreceptor cell as reported for humans [13,15]. *RB1* knockdown experiments on explanted human fetal retina specimens showed that early postmitotic LM-type cone precursor cells are especially vulnerable to pRB loss and are deemed likely cells-of-origin [15,16,17]. It has been suggested that this vulnerability to *RB1* loss depends on the high expression of pRB, MDM2 and MYCN in human cone precursor cells. The availability of human pluripotent stem cells, their potential to differentiate into neural retina and their accessibility to genetic engineering enabled the establishment of human cellular models for retinoblastoma where tumor initiation during retinal differentiation can be monitored. Several protocols have been described for robust neural retina differentiation of human pluripotent stem cells in 3D organoids, employing organoid formation in suspension or via an intermediate adherent step [18,19,20,21,22,23]. Retinal organoids develop all seven cell types of the mature retina with timing and organization similar to that seen in vivo [24]. To date, four studies discussed the differentiation of retinal organoids in modelling retinoblastoma [25,26,27,28]. Three studies focused on in vitro differentiation of *RB1* mutant human pluripotent stem cells into retinal organoids for 90 to 150 days, whereas one study investigated tumor formation of organoid-derived cells of d45 in orthotopic xenograft models. All studies reported consistently on persistent proliferation and changes in retinal cell-type composition. All, except for the study of Zheng et al., showed tumorigenic potential of organoid-derived cells in vitro or in mouse xenograft models. Xenograft tumors showed features of human retinoblastoma. Although some features are described by all studies, some differences remain, which might be due to differences in the genetic background of the cells used, use of similar but different differentiation protocols and discrepancies in analyses applied. Detailed analyses of several retinoblastoma in vitro models will contribute to formation of a consensus in the application of such models.

The present study adds to the previous studies and confirms their in vitro data, contributing to the decision-making process whether retinal organoids could be useful models of retinoblastoma. The data suggest that retinoblastoma originates from immature or maturing cone photoreceptor cells. Differentiation of *RB1*-modified hESCs into retinal organoids revealed loss of structure and overrepresentation of proliferating cone photoreceptors only in organoids derived from hESCs not expressing *RB1* (*RB1*^ko^). Transcriptome analysis of primary retinoblastoma and organoids revealed the development of a retinoblastoma expression signature in *RB1*^ko^ organoids. Expression of proliferation-associated genes was maintained and accompanied by reduced expression of retinal differentiation-associated genes. Gene ontology and enrichment analyses pointed to differences in extracellular matrix organization and the development of a parainflammation signature in late *RB1*^ko^ organoids.

## 2. Materials and Methods

### 2.1. Human Embryonic Stem Cells and Tumor Specimens

All experiments were performed in accordance with relevant guidelines and regulations. Use of human embryonic stem cells (hESCs) has been reviewed and approved by the Central Ethical Review Board for Stem Cell Research at the Robert Koch Institute, Berlin, Germany (Az.3.04.02/0101) and the Ethical Review Board of the University of Duisburg-Essen (16-7215-BO). For patient tumor samples, written informed consent was obtained and use of tumor samples for RNA-seq was reviewed and approved by the Ethical Review Board of the University of Duisburg-Essen (17-7384-BO). Tumor specimens were obtained after primary enucleation, directly frozen and kept at −80 °C until RNA preparation. Samples were chosen based on availability, loss-of-heterozygosity in the tumor (used as measure for absence of normal retina tissue), early age at diagnosis and type of mutation (premature stop codon) in *RB1*. Sole presence of mutant *RB1* transcript was verified by RT-PCR and sequencing.

### 2.2. Generation and Cultivation of hESCs

Cells were cultivated on Matrigel-coated plasticware in StemMACS iPS-Brew XF medium (Miltenyi Biotec, Bergisch Gladbach, Germany) at 37 °C in 5% CO_2_ humidified atmosphere. Cell splitting was performed with Gentle Cell Dissociation Reagent (STEMCELL Technologies, Cologne, Germany) at a ratio of 1:8 every 4–5 days. Human ESCs were genetically modified by CRISPR/Cas9 as described [29,30]. Cell lines H9_RB1ex3_G12LS and H9_RB1ex3_C07, modified in *RB1* exon 3, carry an identical deletion of seven basepairs (LRG_517t1:c.374_380del p.Glu125Valfs*9; GRCh38: Chr13:48342708-48342714) in heterozygous and homozygous states, respectively. Cell lines H9_RB1ex1_8B3 and H9_RB1ex1_8F2 carry deletions of entire *RB1* exon 1 of circa 400 basepairs in heterozygous and compound heterozygous state, respectively (heterozygous: GRCh38: Chr13:48303704-48304100; compound heterozygous: GRCh38: Chr13:48303687-48304103 and GRCh38: Chr13:48303708-48304102).

### 2.3. Differentiation of hESCs into Neural Retina

Organoid differentiation of hESCs into neural retina was performed as described and illustrated in Figure S1 [31]. Differentiations were set up as three independent biological replicates.

### 2.4. Cryosectioning and Immunofluorescent Staining of 3D Organoids and Quantification

Cryosectioning and immunofluorescent staining and signal quantification was performed as described in [31]. Antibodies for immunostaining are listed in Table S1. Images were taken with a Leica SP8 confocal laser scanning microscope. Quantification was performed as described, but with an adapted macro for three channel images (Supplementary File 1). Wilcoxon rank sum test was applied to test for significance with * *p* ≤ 0.05, ** *p* ≤ 0.01, *** *p* ≤ 0.001, **** *p* ≤ 0.0001. Boxplots were generated using ggplot2 with default settings.

### 2.5. RNA-seq

Organoids were harvested at d35, d96 and d152 of differentiation and total RNA was isolated using Monarch Total RNA Miniprep kit (NEB, Frankfurt, Germany). Where possible, equal amounts of RNA of three organoids per experiment were pooled (for exceptions, see Table S2) and sequenced using the TruSeq Stranded mRNA kit (Illumina, Berlin, Germany) by CeGaT, Tübingen, Germany. Paired reads were quality controlled using FastQC (v0.11.9; https://www.bioinformatics.babraham.ac.uk/projects/fastqc/, accessed on 23 September 2020) and trimmed via fastq-mcf (ea-utils, 1.05). Trimmed reads were aligned to the GENCODE genome release 35 (GRCh38.p13) via STAR (2.7.5c) and mapped reads counted via htseq-count (0.13.0; -q -m intersection-nonempty --stranded = reverse –r;) [32,33]. Samtools served for conversion/sorting of sam and bam files (0.1.19) [34]. Normalization and further analyses were performed in the R environment (4.0.2; www.r-project.org, accessed on 14 April 2022) including DESeq2 (1.30.0) enriched by ENSEMBL gene annotations (v101) [35]. Batch effects in the gene expression model were corrected and FDR-corrected *p*-values calculated. R-packages clusterProfiler (3.18.0) and enrichplot (1.10.1) were used to construct heatmaps [36]. GOSemSim (2.16.1) was used for reduced redundancy GO term enrichment analysis of differentially expressed genes and gene set enrichment analysis performed via GSEA (v4.1.0) using default settings and the Hallmark gene set of MSigDB (h.all.v7.2.symbols.gmt) on normalized gene expression data of d152/d160 organoid samples for genes with minimal expression of 10 reads [37,38]. Datasets were normalized based on DESeq2, before and after exclusion of the three outlier samples G12LS_30DK. Otherwise, samples shown in the same figure were normalized against each other and analyzed using DESeq2 before plotting. For representation in heatmaps, *p*-values and log2FC (indicated with the respective figures) were chosen such that each heatmap displayed a comparable number of genes. Since gene expression changes between genotypes at d152 were the main point of interest, DEGs retrieved in multiparametric analysis) were filtered with *p* < 0.05 and log2FC > 1. Log2FC plotted for the retinoblastoma expression signature was calculated as follows: ratio 1 = *RB1*^wt/het^_d152/*RB1*^wt/het^ _d35, ratio 2 = *RB1*^ko^_d152/*RB1*^ko^_d35, log2FC = ratio 2/ratio1. 

## 3. Results

### 3.1. Differentiation into Neural Retina Is Impaired in RB1^ko^

*RB1* mutant H9 human embryonic stem cells were generated and subsequently differentiated into retinal organoids. The clonal sublines H9_RB1ex3_G12LS and H9_RB1ex3_C07 were generated after CRISPR/Cas9 editing of *RB1* and carry identical deletions of seven basepairs in *RB1* exon 3 in both heterozygous and homozygous state, respectively. Absence of pRB in H9_RB1ex3_C07 has been confirmed (Figure S2) [29]. Genome integrity of the cell lines was analyzed by whole genome sequencing and revealed 19 and 21 variants private to H9_RB1ex3_G12LS and H9_RB1ex3_C07, respectively (Figure S3, Supplementary Methods, Supplementary File 2, Supplementary File 3). None of these variants coincided with a predicted CRISPR/Cas9 off-target site (Table S3), indicating random and independent acquisition during cell cultivation. The H9 hESC line used here, and consequently the two sublines, carry a heterozygous D281N mutation in *TP53* (GRCh38/hg38, chr17:7673779 C > T), a variant deemed pathogenic (https://www.ncbi.nlm.nih.gov/clinvar, accessed on 30 April 2021).

**Figure 1:**
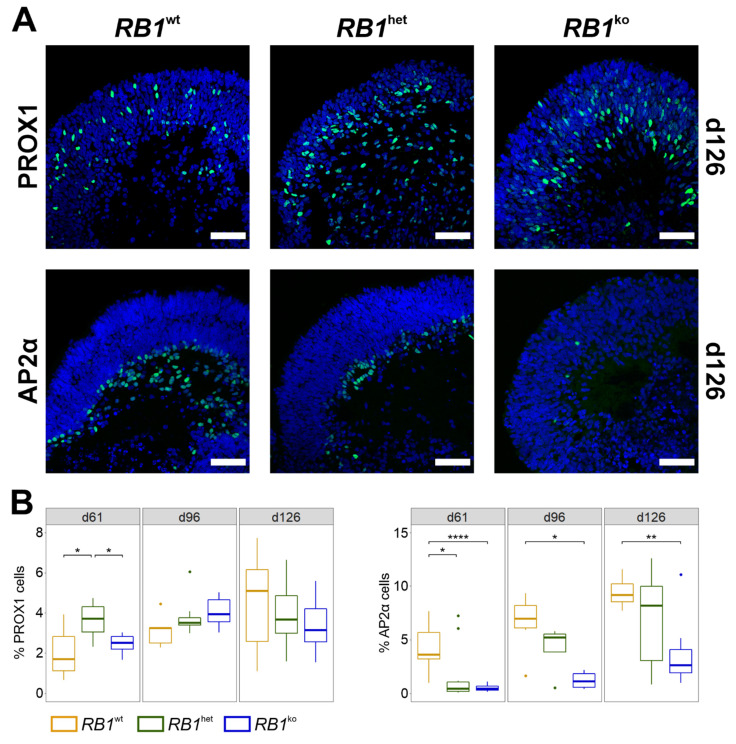
Amacrine and horizontal cells in differentiating retinal organoids. (**A**) Representative images of immunofluorescent staining of organoids at d126 for marker proteins PROX1 (horizontal cells) and AP2α (amacrine cells) (green). Nuclei were counterstained with DAPI (blue). Scale bar 50 μm. (**B**) Quantification of PROX1- and AP2α-positive cells based on microscopy images. Percentages of marker-positive cells per total DAPI-positive area are given. * *p* ≤ 0.05, ** *p* ≤ 0.01, **** *p* ≤ 0.0001.

Retina differentiation and development of retinal cell types in 3D organoids were compared between parental H9 hESCs and clonal sublines H9_RB1ex3_G12LS and H9_RB1ex3_C07 [31]. Organoids of the respective genotypes are designated *RB1*^wt^, *RB1*^het^ and *RB1*^ko^ throughout the manuscript. Morphologically, development of retinal tissue was observed from d12 onward (Figure S1A). Success of organoid formation, size and formation of retinal loops were comparable between the genotypes up to d126. However, at d152, *RB1*^ko^ organoids were smaller and surrounded by shed cells, while the outer retinal layer appeared to be absent in brightfield microscopy (Figure S1B). Immunofluorescent staining was used to determine the appearance of the seven retinal cell types (ganglion, horizontal, amacrine, rod and cone photoreceptors, bipolar and Müller glia) during differentiation. Induction of the eye field was verified at d35 by presence of retinal progenitor cells, staining positive for PAX6, RX, VSX2 and early photoreceptor cells, staining positive for CRX (Figure S4). Ganglion cells expressing BRN3 isoforms (encoded by *POU4F1*, *POU4F2* and *POU4F3*) were observed at d35 in the inner part of organoids and their number appeared similar in all types of organoids (Figure S5). Horizontal and amacrine interneurons developed from d61 onward and located in the middle and innermost layers of organoids, respectively (Figure 1A). Based on the quantification of PROX1-positive cells at d126, numbers of horizontal cells were not significantly different between the three genotypes (Figure 1B). The numbers of amacrine cells (AP2α) at d126 correlated with *RB1* genotype: 10% in *RB1*^wt^, 7% in *RB1*^het^ and only 3% in *RB1*^ko^ (Figure 1B), which was also described by Rozanska et al. [27].

**Figure 2:**
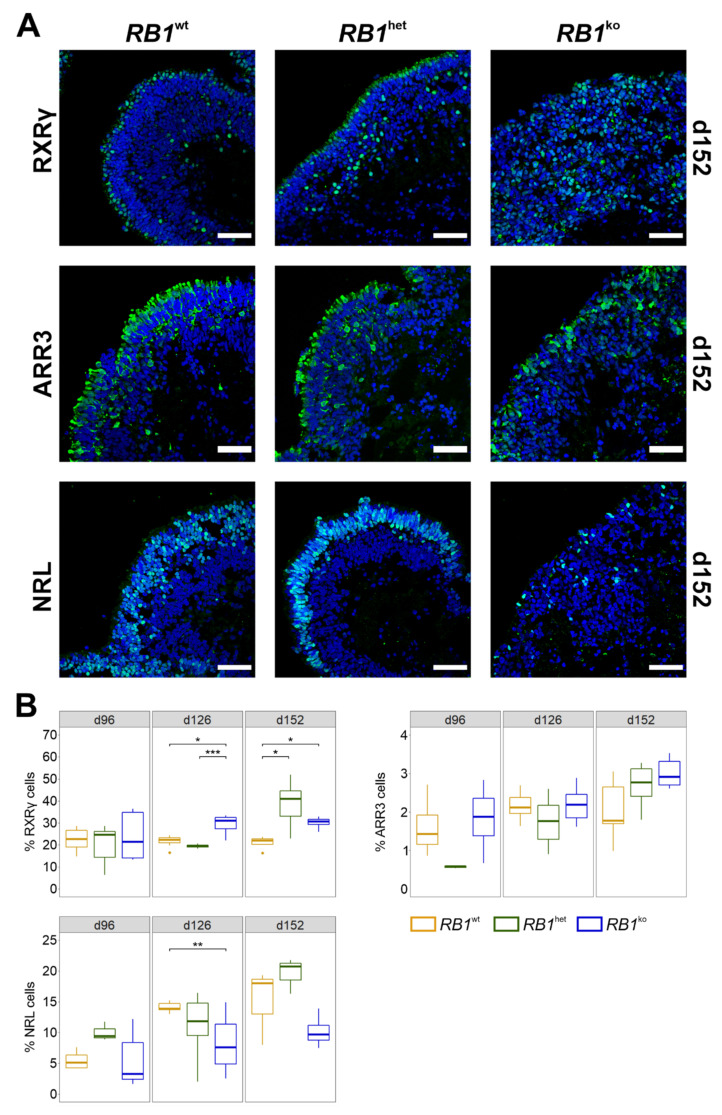
Cone and rod photoreceptors at d152. (**A**) Representative images of immunofluorescent staining of cells expressing RXRγ (immature cone marker), ARR3 (maturing cone marker), NRL (early rod marker). Marker proteins in green, nuclei counterstained with DAPI (blue). Scale bar 50 μm. (**B**) Quantification of RXRγ- and ARR3-positive cone photoreceptors and NRL-positive rod photoreceptors based on microscopy images. Percentages of marker-positive cells per total DAPI-positive area are given. * *p* ≤ 0.05, ** *p* ≤ 0.01, *** *p* ≤ 0.001.

Bipolar and Müller glia cells arose late during differentiation. Bipolar cells (PKCα, VSX2) located in the middle layer of the organoid in *RB1*^wt^ and *RB1*^het^ but were absent in *RB1*^ko^ (Figure S5). Müller glia cells (VIM, SOX9, SOX2) interconnect all retinal layers and their abundance was reduced in *RB1*^ko^, compared to *RB1*^wt^ and *RB1*^het^ (Figure S5). Rod and cone photoreceptors are the light-sensitive cells of the retina and markers for proper retinal differentiation in vitro. They started to develop around d90 of differentiation. In *RB1*^ko^, photoreceptors did not form a highly organized cell layer at the outer rim of the organoid, which might correlate with the observed difficulty of detecting a visible retinal layer in brightfield images (Figure 2A and Figure S1B). Developing and mature rod photoreceptors (NRL, RHO) were less abundant in *RB1*^ko^ when compared to *RB1*^wt^ and *RB1*^het^ specimens (Figure 2 and Figure S6). Immature cone photoreceptors (RXRγ) were detectable at d152 in reasonable and stable numbers and enriched in *RB1*^ko^ (Figure 2). Maturing cone photoreceptors (ARR3) developed up to d152 and numbers did not differ between the three genotypes (Figure 2).

In summary, retinal differentiation resulted in formation of all seven retinal cell types in *RB1*^wt^ and *RB1*^het^ organoids. In *RB1*^ko^, layered organization of the retina was lost and, while differentiation of most retinal cell types was inefficient, immature RXRγ-positive cone photoreceptor cells developed abundantly.

### 3.2. Persistent Proliferation in RB1^ko^

**Figure 3:**
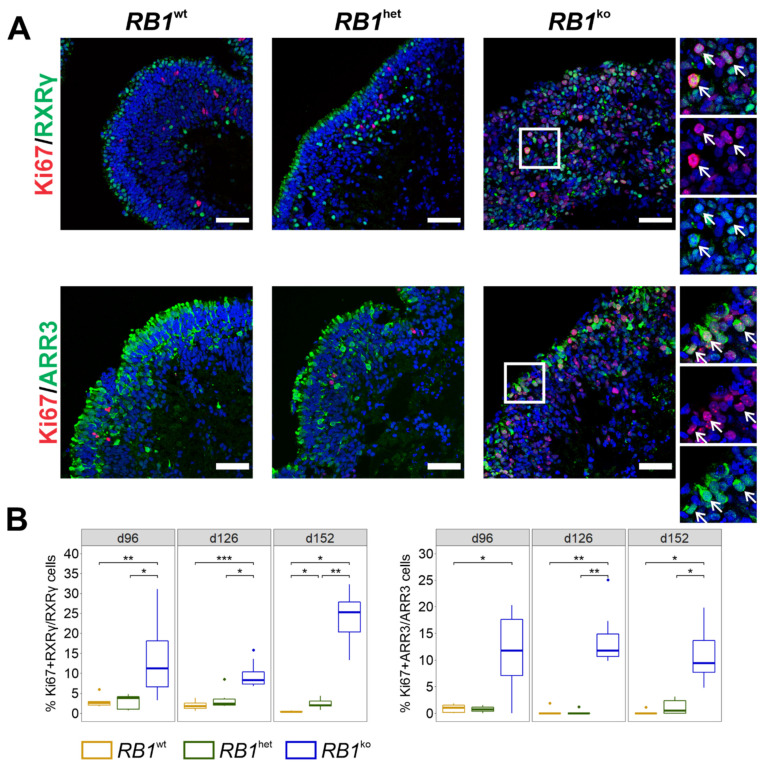
*RB1*^ko^ organoids develop proliferating RXRγ- or ARR3-positive cone photoreceptors. (**A**) Representative images of co-immunofluorescent staining of cone marker proteins RXRγ and ARR3 (green) with Ki67 (red) at d152 of differentiation. Arrows indicate double-positive cells. Nuclei were counterstained with DAPI (blue). Scale bar 50 μm. (**B**) Quantification of cells staining positive for cone marker proteins and Ki67. The percentage of double-positive area normalized to cone marker-positive area is given. * *p* ≤ 0.05, ** *p* ≤ 0.01, *** *p* ≤ 0.001.

To analyze whether the observed gain in RXRγ-positive cone photoreceptor cells was a result of their excess proliferation, co-staining of proliferation marker Ki67 with cone marker proteins was performed (Figure 3A). With ongoing differentiation, numbers of proliferating cells (Ki67) decreased in *RB1*^wt^ and *RB1*^het^ but represented 15–20% of all cells in *RB1*^ko^ at d152 (Figure S7). Of the cells expressing RXRγ or ARR3, 11–25% and 10–12%, respectively, were also positive for Ki67 (Figure 3B).

**Figure 4:**
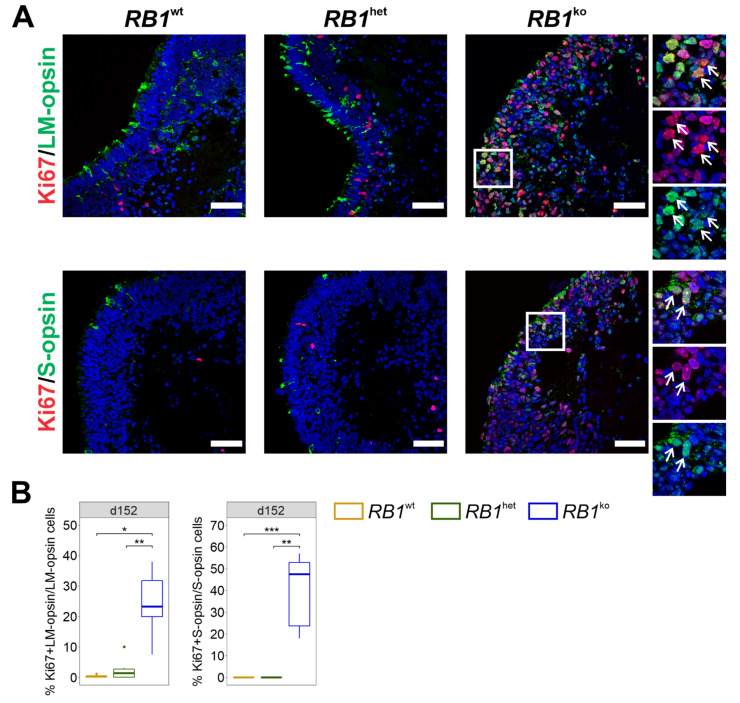
*RB1*^ko^ organoids exhibit proliferating LM- or S-opsin-positive photoreceptors. (**A**) Representative images of co-immunofluorescent staining of cone marker proteins LM-opsin or S-opsin (green) with Ki67 (red) at d152 of differentiation. Arrows indicate double-positive cells. Nuclei were counterstained with DAPI (blue). Scale bar 50 μm. (**B**) Quantification of cells staining positive for cone marker proteins and Ki67. The percentage of double-positive area normalized to cone marker-positive area is given. * *p* ≤ 0.05, ** *p* ≤ 0.01, *** *p* ≤ 0.001.

LM-type cone photoreceptors are reportedly especially sensitive to loss of pRB [16,17]. Staining for LM-opsin and S-opsin revealed overall few positive cells with no significant difference in percentage in all three genotypes (Figure S8). Cells staining positive in *RB1*^ko^ show less mature photoreceptor morphology, more nuclear than cytoplasmic localization and lower overall signal intensity when compared to staining in *RB1*^wt^ and *RB1*^het^ (Figure S8). Co-staining of LM-opsin and S-opsin with Ki67 was observed in *RB1*^ko^ only (Figure 4). In comparison, co-staining of Ki67 was not detected in Müller glia cells (VIM) and bipolar cells (PKCα), observed very rarely in rod photoreceptors (NRL) and seen more often in horizontal cells (PROX1) (Figure S9). In summary, cone photoreceptors, identified by expression of RXRγ, ARR3, LM-opsin or S-opsin, were the main proliferating cells in *RB1*^ko^ organoids.

### 3.3. RNA-seq Confirmed Aberrant Retinal Differentiation and Proliferation in RB1^ko^

**Figure 5:**
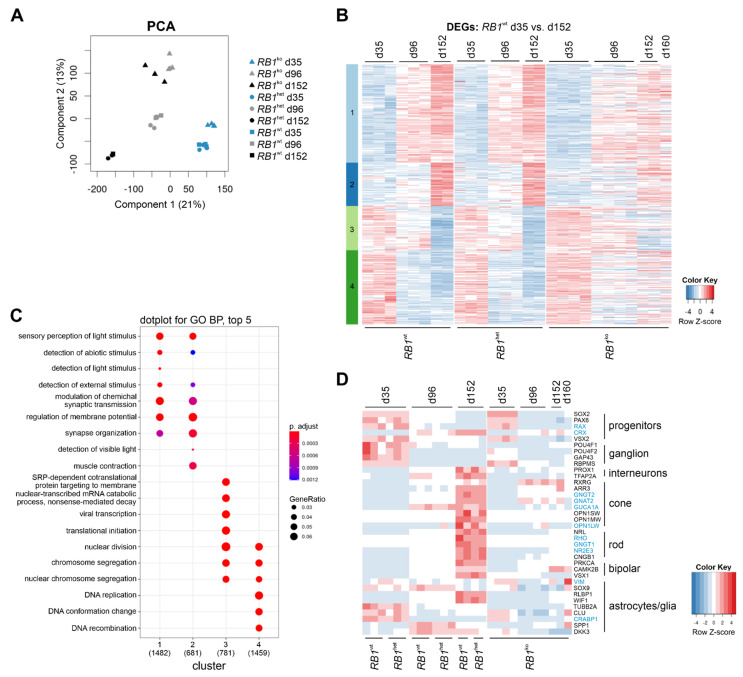
Transcriptome analysis revealed impaired retinal differentiation in *RB1*^ko^ after d35. (**A**) Principal component analysis. Color: time point (blue d35, grey d96, black d152), symbols: genotype (triangle: *RB1*^ko^, dot: *RB1*^het^, square: *RB1*^wt^, filled: H9_RB1ex3, open: H9_RB1ex1). (**B**) DEG analysis of *RB1*^wt^ d35 versus d152 using log2(FC) > 1, *p*-adjust < 0.05 for filtering. Heatmap shows 7254 genes clustered into c1 to c4. Expression of the identified DEGs in *RB1*^het^ and *RB1*^ko^ was plotted. (**C**) Top 5 (*p*-adjust < 0.05) enriched biological processes per cluster determined by reduced redundancy GO analysis. The total number of DEGs in each cluster associated with a GO-term is given in parentheses, dot size: ratio of number of DEGs present in the specific GO-term versus total number GO-term associated DEGs in this cluster, dot color: *p*-adjust. If any of the top 5 terms of one cluster is present in another cluster, corresponding dots are plotted. (**D**) Gene expression of marker genes for retinal differentiation (normalized counts, scaling per row). Black/blue: genes identified/not identified as DEGs in multiparametric analysis (Figure 6).

To relate the observed phenotype of *RB1*^ko^ to changes in gene expression, transcriptomes of organoids were generated. To validate and strengthen the obtained results, organoids heterozygous or homozygous for deletion of the entire exon 1 of *RB1* were included in transcriptome analysis (Table S2) [30]. Analysis of such organoids showed absence of pRB and expression of retinal markers comparable to organoids with modifications in *RB1* exon 3 (Figures S2 and S10–S12). Principal component analysis (PCA) showed separation of *RB1*^wt^ and *RB1*^het^ samples by day of differentiation (Figure 5A). *RB1*^ko^ samples clustered separately from *RB1*^wt^ and *RB1*^het^ and did not separate between d96 and d152. Based on the PCA plot, we concluded that the type of mutation had no influence on differentiation, because the *RB1*^ko^ sample groups did not split according to the *RB1* mutation whether involving exon 3 or exon 1 modifications. Of note, results of *RB1*^het^ samples of one differentiation experiment were classified as outliers and excluded from further analyses (Figure S13, Table S2).

**Figure 6:**
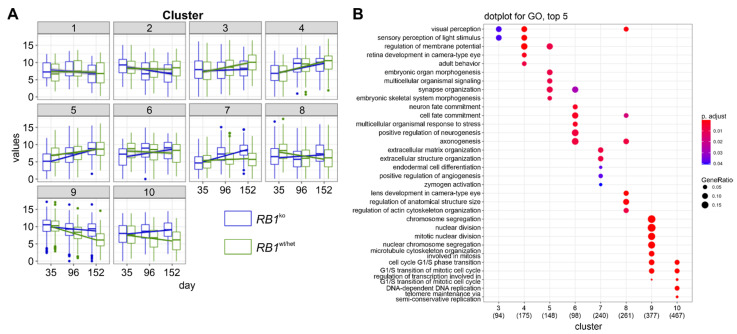
Multiparametric analysis of differential gene expression in retinal differentiation. (**A**) Multiparametric analysis considering genotype (*RB1*^wt/het^ versus *RB1*^ko^), day of differentiation and batch effects of independent experiments. DEGs were filtered by *p*-adjust < 0.05 and log2FC > 1. Filtered DEGs were assigned to 10 clusters. Expression of genes in clusters over time is shown. (**B**) Top 5 (*p*-adjust < 0.05) enriched biological processes per cluster determined by reduced redundancy GO analysis. The total number of DEGs in each cluster associated with a GO-term is given in parentheses, dot size: ratio of number of DEGs present in the specific GO-term versus total number of GO-term associated DEGs in this cluster, dot color: *p*-adjust. If any of the top 5 terms of one cluster is present in another cluster, corresponding dots are plotted.

In a first analysis, changes in gene expression between d35 and d152 of retinal differentiation were determined in *RB1*^wt^ (Figure 5B). The obtained differentially expressed genes (DEG) were assigned to four clusters. Genes upregulated during differentiation were associated with GO terms (biological processes) sensory perception of light and synaptic transmission; downregulated genes were enriched for cell division and mRNA processing (Figure 5C). This reflects induction of retina differentiation accompanied by ceasing proliferation. No substantial differences in gene expression were evident between *RB1*^het^ and *RB1*^wt^, confirming that heterozygous inactivation of *RB1* has no effect on retinal differentiation. In *RB1*^ko^, gene expression deviated after d35: genes involved in retina differentiation were not upregulated, while those affecting DNA replication and proliferation were not downregulated (Figure 5B). Plotting expression of specific retinal marker genes confirmed the results obtained by immunofluorescence and highlighted the lack of retinal differentiation in *RB1*^ko^ after d35 (Figure 5D). Only the markers for cone photoreceptors *RXRG* and *GNAT2* were markedly upregulated in *RB1*^ko^ in later stages of differentiation. On the other hand, expression of several cell-cycle regulators and the pRB-regulated E2F transcription factors remained high in *RB1*^ko^ (Figure S14). In a second analysis, the influence of parameters time and genotype on gene expression was determined. To simplify analysis, *RB1*^wt^ and *RB1*^het^ were combined in one genotype since immunostaining and gene expression patterns look alike. Differences between *RB1*^wt/het^ and *RB1*^ko^ in gene expression patterns were observed in five clusters (Figure 6A, clusters 3, 6, 7, 8, 10).

As already described, genes associated with visual perception (c3) became upregulated in *RB1*^wt/het^ but not in *RB1*^ko^, and genes needed for G1/S transition of mitotic cell cycle (c10) were upregulated in *RB1*^ko^ but downregulated in *RB1*^wt/het^ (Figure 6B and Figure S15). Additionally, genes related to cell fate commitment and neurogenesis (c6) and development of anatomical structure size (c8) were active at d35 in *RB1*^wt/het^ but only at d152 in *RB1*^ko^, illustrating the delay in differentiation in *RB1*^ko^. Interestingly, genes clustered in c7 were strongly upregulated in *RB1*^ko^ only and were enriched in the process extracellular matrix organization. This might be related to the observed disintegration of *RB1*^ko^ organoids after d126 of differentiation (Figure S1B). GSEA analysis of expression data of d152 delivered similar results with prominent enrichment of gene sets E2F_targets, MYC_targets, mitotic_spindle and epithelial-mesenchymal transition (Figure S16). In addition, gene sets related to immune pathways were highlighted.

Gene expression profiles of the retinal organoids were compared to publicly available data. DEG and cluster analysis between fetal and adult retina revealed that organoid differentiation mimics retina development in vivo: the transition of gene expression in clusters c1, c4 and c5 over time was similar to the shift of gene expression from fetal retina to adult retina (Figure S17). The DEG expression pattern determined in *RB1*^wt^ (Figure 5) was comparable to that obtained in the retinal organoid study reported by Kaya et al. (Figure S18) [39].

### 3.4. RB1^ko^ Organoids Gain Retinoblastoma Expression Signature

**Figure 7:**
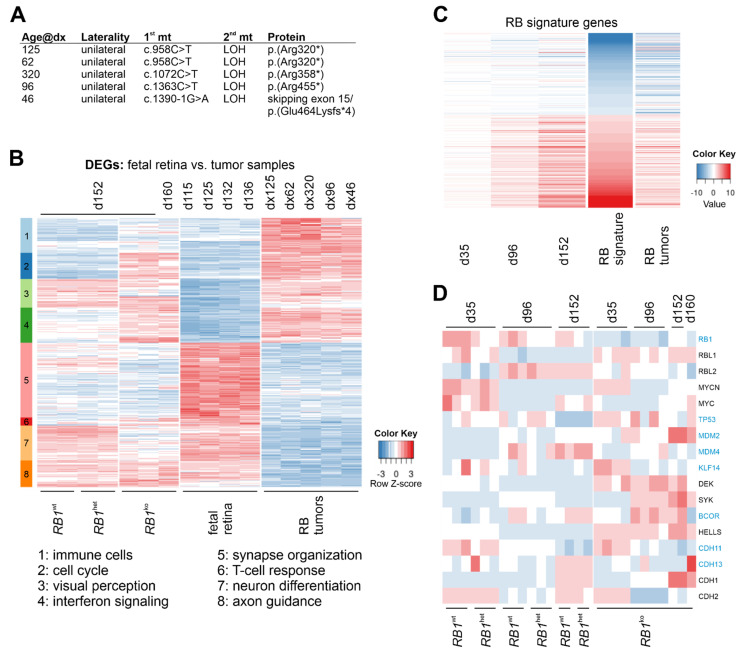
*RB1*^ko^ gain retinoblastoma signature during differentiation. (**A**) Data of tumor samples sequenced. Age@dx: age at diagnosis in days after birth; 1st mt/2nd mt: first/second mutation in tumor (note: c.1 is A of ATG start codon of LRG_517t1). * indicates generation of translational stop codon. (**B**) DEGs were determined between fetal retina and retinoblastoma samples (*p* < 0.001, log2(FC) > 2). Expression of these genes in organoids at d152/d160 was plotted. Cluster analysis retrieved eight clusters. (**C**) Expression of 510 retinoblastoma signature genes in *RB1*^ko^. Log2(FC) values of the DEGs in *RB1*^ko^ were normalized to log2(FC) values in *RB1*^wt^ samples at d35, d96 and d152 of differentiation; expression in the five tumor samples was normalized to public data for fetal retina. (**D**) Gene expression (normalized counts, scaling per row) of genes associated with retinoblastoma as observed in retinal organoids and tumor samples. Black/blue: genes identified/not identified as DEGs in multiparametric analysis (Figure 6).

To determine whether gene expression of *RB1*^ko^ organoids resembles that of retinoblastoma, transcriptomes of five tumor specimens were generated. Tumor samples were selected based on young age at diagnosis, mutation in *RB1* in a 5′-exon and presence of LOH in the tumor (Figure 7A). Public data for fetal retina at days 115, 125, 132 and 136 post conception were used for comparison, because gene expression at this stage appeared most similar to organoids at d152 [40,41]. DEGs between fetal retina and tumor samples were determined and after clustering, eight clusters were evident (Figure 7B, Figure S19). Genes in c1 to c4 were upregulated in tumor samples but weakly expressed in developing retina. Reduced redundancy GO term analysis revealed terms associated with immune regulation (c1, c4), cell-cycle progression (c2) and visual perception (c3). Genes in clusters c5 to c8 were expressed in fetal retina but not in tumor samples. GO analysis revealed enrichment for processes involved in neuronal differentiation (c5, c7, c8). The small cluster c6 connoted T-cell-mediated immune regulation. Organoids *RB1*^wt^ and *RB1*^het^ at d152 were more similar to fetal retina (c1, c2, c4, c7 and c8) than to tumor samples, showing weak expression in immune and cell cycle-related clusters (c1, c2, c4) but prominent expression in processes of neuronal differentiation (c7, c8). In contrast, *RB1*^ko^ at d152 were more similar to tumor samples with enhanced expression in clusters related to cell cycle (c2), visual perception (c3) and type I interferon response (c4) but low expression of genes related to neuronal differentiation (c5, c7). A retinoblastoma gene expression signature was distilled from data of two earlier studies and transcriptome data of *RB1*^ko^ at d152 and the tumor samples were compared to this signature [5,6]. In the study by Ganguly et al., gene expression in six tumors was compared to that of normal retina, whereas Kapatai et al. compared 21 tumors to publicly available expression data from fetal and adult retina. The gene set common to these two earlier studies consisted of 510 members, 240 down- and 270 upregulated in retinoblastoma when compared to normal retina. We analyzed the expression of these DEGs in *RB1*^ko^ and tumor samples. The gene expression signature of the tumor samples confirmed that of the two earlier studies (Figure 7C).

In *RB1*^ko^ organoids, the similarity to the retinoblastoma signature increased from d35 to d152 of differentiation. Plotting the expression of genes known to be associated with retinoblastoma development, such as *MDM2*, *DEK*, *SYK* and *HELLS*, confirmed their upregulation in *RB1*^ko^ when compared to *RB1*^wt^ and *RB1*^het^ and in the tumor specimens (Figure 7D). Expression of *RB1* mRNA was highest in *RB1*^wt^, intermediate in *RB1*^het^ and lowest in *RB1*^ko^. The two other members of the pocket protein family, *RBL1* (p107) and *RBL2* (p130), showed distinct expression changes during retinal differentiation: in *RB1*^wt^ and *RB1*^het^, *RBL1* was expressed at d35 only, whereas *RBL2* became upregulated from d96 onward. In *RB1*^ko^, *RBL1* was not downregulated upon differentiation and *RBL2* not upregulated. This reflects the expression pattern observed in native retinoblastoma with prominent expression of *RBL1* but almost undetectable expression of *RBL2*, further reinforcing the validity of the retinal organoid model [17].

## 4. Discussion

We present *RB1*-modified 3D retinal organoids as a model system for in vitro analysis of retinoblastoma. *RB1*^wt^ and *RB1*^het^ organoids were indistinguishable in retinal differentiation by morphology, immunofluorescent staining and global RNA expression. In contrast, while in *RB1*^ko^ organoids differentiation was initiated, proper development of retinal cell types was impaired. Instead, proliferation, specifically of cone photoreceptors, persisted. Lack of differentiation and persistent proliferation was accompanied by loss of structure in *RB1*^ko^ organoids and their disintegration after d126. Transcriptome profiles revealed gradual development of a retinoblastoma expression signature in *RB1*^ko^ with upregulation of proliferation-associated genes and retinoblastoma-related oncogenes such as *DEK*, *SYK* and *HELLS*.

CRISPR/Cas9 edited cell lines were analyzed for off-target effects by whole genome sequencing (WGS, Figure S3). Of 58 single nucleotide variants detected in the edited cell lines, 18 were shared between H9_RB1ex3_G12LS and H9_RB1ex3_C07 cell sublines. This could indicate the presence of low-level subclones in the parental hESC line H9, which went undetected in global WGS, and only became apparent through the establishment of clonal edited cell lines. None of the acquired variants was related to predicted off-target sites of the sgRNA used for editing. A missense variant in *TP53* (p.D281N), hitherto unreported in H9 hESCs, was present with variant allele fractions of 43% in H9 and H9_RB1ex3_G12LS and 55% in H9_RB1ex3_C07. This indicates fixation of the mutant allele in a heterozygous state in all three cell lines. This variant has been classified as pathogenic (https://www.ncbi.nlm.nih.gov/clinvar, accessed on 30 April 2021). Recurrent acquisition of *TP53* mutations in hESCs reportedly confers growth advantage [42].

In organoid differentiation, *RB1*^ko^ showed persistent proliferation, delayed differentiation and loss of structure. These observations are in line with the role of pRB as negative cell-cycle regulator and RNA-seq analyses confirmed upregulation of E2F-transcription factors and their target genes (Figures S14–S16) [43]. The enrichment of genes associated with MYC-targets might indicate that deregulation of gene expression by *MYCN* could be an important factor in retinoblastoma development, strengthened by rare retinoblastoma entities in which *MYCN* amplification substitutes for mutational *RB1* inactivation [4]. Accordingly, the MYC-target gene *PLK1* was one of the top upregulated genes in *RB1*^ko^, which fits the anti-proliferative effect of PLK1-inhibition in retinoblastoma cell lines [9].

Several stem-cell models with engineered biallelic inactivation of *RB1* have been described [25,26,27,28,44,45]. In our study, as well as in those previous studies, *RB1* loss did not alter stem-cell properties or cell-cycle kinetics in undifferentiated stem cells, nor did aberrant karyotypes appear. Including this one, five studies describe differentiation of *RB1* mutant human pluripotent stem cells into retinal organoids [25,26,27,28]. Norrie et al. used iPSCs of patients carrying a germline heterozygous mutation in *RB1* and differentiated them in vitro up to d45 before injecting them into the vitreous of mouse eyes [26]. For tumor formation, inactivation of the second allele of *RB1* needed to occur in the xenografts, which was observed with low frequency only. However, the tumors that developed resembled retinoblastoma based on gene expression, DNA methylation and morphology. This model might be the one that most resembles retinoblastoma formation in humans, but it is rather inefficient and costly. Clean in vitro models of retinoblastoma development might be preferable for studying certain aspects. In our study and the ones by Liu et al., Rozanska et al. and Zheng et al., H9 hESCs modified in *RB1* were differentiated into neural retina for up to 150 days in organoid cultures in vitro [25,27,28]. Modifications in *RB1*, differentiation protocols and types of analyses differed between the studies. Still, increased proliferation and reduced numbers of other retinal cell types was a common finding. Applying staining for photoreceptor markers, it could be shown that predominantly cone precursors (RXRγ) and maturing cone photoreceptors (ARR3) were proliferating in *RB1* mutant organoids, which was confirmed by single-cell RNA-seq by Liu et al. and Rozanska et al. (Figure 3 and Figure 4) [25,27]. This gain of proliferating cone photoreceptors was accompanied by a reduction of amacrine and bipolar cells especially, as well as rod photoreceptors, suggesting a need for pRB in the differentiation into these retinal cell types (Figure 1, Figure 2 and Figure S6) [25,27]. Expression studies also showed the development of a retinoblastoma signature and upregulation of specific genes associated with retinoblastoma in late *RB1* mutant organoids (Figure 7D and Figure S14) [25,27], since the deviation of differentiation in *RB1* knockout organoids became evident only after about 40 days (Figure 5B) [25]. Disintegration of organoids and shedding of cells at later stages of differentiation was observed by us and Liu et al., who could further show that injection of the shed cells resulted in the development of tumors in mice (Figure S1B) [25]. Tumorigenic potential of cells derived by organoid differentiation of *RB1* mutant stem cells was shown using different in vitro and xenograft approaches [25,26,27]. Taken together, all these studies present evidence for proliferating cone photoreceptors as likely cells-of-origin of retinoblastoma and the potential of retinal organoid differentiation in retinoblastoma research, as exemplified by their use in drug-testing strategies in two of the studies [25,27].

Cone photoreceptors of the LM-type have been described as sensitive to *RB1* loss, reacting by re-entry into the cell cycle [17]. We detected Ki67 expression both in S- or LM-opsin-positive cone photoreceptor cells, but only in *RB1*^ko^ (Figure 4). In the developing retina, S-type cones develop first, while LM-type cones are supposed to develop from this population by a shift in opsin expression. In the transition period, co-expression of S- and LM-opsin has been described [46]. In native retinoblastoma, expression of LM-opsin was dominant, while S-opsin was expressed only rarely in conjunction with LM-opsin [15]. The organoid model might indicate that also S-opsin expressing cone cells could serve as cells-of-origin of retinoblastoma, later switching to LM-opsin expression.

The presence of photoreceptor cells in retinoblastoma has long been under debate since tumors contain rosette structures suggestive of primitive photoreceptors and clinical diagnosis is based on positivity for the photoreceptor marker CRX and the proliferation marker Ki67 [47]. The formation of rosette structures in certain areas of *RB1*-negative organoids was described [25,27]. Staining for CRX was strongly positive at d35 in *RB1*^ko^, consistent with the findings in retinoblastoma (Figure S4). In 2009, Xu et al. suggested that retinoblastoma shows properties of cone photoreceptors but not other retinal cell types [15]. This is consistent with a lack or reduced development of rods, amacrine, bipolar and Müller glia cells in *RB1*^ko^ (Figure 1, Figure 2, Figures S5 and S6) [25,27]. Subseqeunt studies supported the notion that ARR3-positive maturing cone photoreceptor cells are especially sensitive to loss of *RB1* expression, reacting with re-entry into cell cycle and development of retinoblastoma [16,17]. Cells positive for ARR3 and Ki67 could be detected only in *RB1*^ko^ organoids, supporting the link between loss of *RB1* and proliferative ARR3-positive cone photoreceptors (Figure 3). Expression studies reported progenitor- and photoreceptor-related gene expression signatures in retinoblastoma and could not clarify the cells-of-origin debate [6,8]. Marker gene expression of more than one retinal cell type was also described in single-cell RNA-seq studies performed on retinal organoids [25,27]. Follow-up studies rather suggested the presence of two subtypes in retinoblastoma, differing in the degree of photoreceptorness [7,48]. Using a multi-omics approach, retinoblastoma was classified into two subtypes: Subtype 1 retinoblastoma are associated with younger age at diagnosis, heritable disease, low rate of genetic aberrations other than *RB1* and a higher grade of photoreceptor differentiation. In contrast, subtype 2 retinoblastoma are, on average, diagnosed at older age, carry the known retinoblastoma-associated chromosomal aberrations, exhibit a less differentiated phenotype and more often result in metastasis [48]. Based on RNA-seq data, subtype 1 tumors are associated with GSEA hallmarks immune and interferon response, photoreceptor differentiation and fatty acid metabolism, whereas subtype 2 tumors show enrichment of genes in hallmarks E2F and MYC genes, cell cycle and neuron differentiation. Comparing the data of *RB1*^ko^ organoids, hallmarks of both subtypes were identified (Figure S16), indicating that at this stage organoids cannot be clearly assigned to one or the other subtype of retinoblastoma.

Our study demonstrates that retinal organoids derived from the *RB1* mutant pluripotent stem-cell model certain aspects of retinoblastoma in vitro. However, some limitations remain, such as the disintegration of *RB1*^ko^ organoids after d126 of differentiation. Since disintegration was not observed in *RB1*^wt^ and *RB1*^het^ samples differentiated in the same experiments, it must relate to a lack of pRB. GO term analysis and GSEA revealed enrichment of genes associated with endothelial–mesenchymal transition (EMT) and immune response specifically in *RB1*^ko^ (Figures S15 and S16). Since the presence of cytokine-secreting immune cells in retinal organoids is implausible and mRNA expression of interferons or interleukins was not detected, these gene signatures may reflect parainflammation [24,49,50]. Parainflammation is described as cell-autonomous, low-grade inflammation without immune-cell infiltrate and a low secretory component mediated by the interferon pathway [49]. The upregulation of genes associated with EMT might relate to changes in the extracellular matrix that could be cause or consequence of inflammatory processes [50]. Parainflammation can be triggered by DNA damage, persistent cell stress or senescence. Of the 40 genes signifying parainflammation, 12 were significantly upregulated in *RB1*^ko^ but not in *RB1*^wt^ or *RB1*^het^ (Figure S20). Given the manifold functions of pRB, the connection between *RB1* loss and parainflammation remains unclear, but *RB1*^ko^ organoids open a possibility for further studies in this direction. Another deficit of our study is that the nature of the cells shed by *RB1*^ko^ after d126 was not addressed. However, Liu et al. also observed shedding of cells in organoids lacking pRB and could show that these cells are able to form tumors in orthotopic xenograft models [25]. Since the analysis of the shed cells by molecular methods and their ability of in vitro or in vivo tumorgenicity is important in a valid retinoblastoma model, such analyses need to be included in future experiments. By immunofluorescence, expression of S- and LM-opsin in retinal organoids was restricted to only a few cells with no significant differences between the genotypes. However, the RNA-seq data clearly indicate upregulation of the opsin genes *OPN1SW*, *OPN1MW* and *OPN1LW* at d152 of differentiation in *RB1*^wt^ and *RB1*^het^, but not in *RB1*^ko^, which is consistent with the delay of retina differentiation in the latter (Figure 5D). Why the observed mRNA upregulation does not correlate with an increase of cells staining positive for these markers in *RB1*^wt^ and *RB1*^het^ remains unclear. Rozanska et al. reported a similar low overall percentage of cells staining positive for S- or LM-opsin in wildtype organoids but indicated a decrease of S- or LM-opsin-positive cells in *RB1*-null organoids [27]. Further experiments using retinal organoids are needed to clarify this issue.

## 5. Conclusions

Biallelic mutation of *RB1* in hESCs and their subsequent differentiation into retinal organoids resulted in aberrant differentiation and enhanced proliferation of cone photoreceptor cells especially, in vitro. The gene expression signature of these organoids became increasingly similar to native tumor material over time. These features were not observed in differentiating wildtype hESCs or hESCs carrying the *RB1* mutation only on one allele. Our findings support the analyses performed in previous studies using retinal organoid differentiation of *RB1* mutant human pluripotent stem cells. This provides good evidence that organoids can serve as useful in vitro models for human retinoblastoma as they exhibit some of the morphological, cellular and molecular markers of the tumor. Moreover, the possibility of time-course analyses and experimental manipulation further strengthens their use in future retinoblastoma research.

## Data Availability

The publicly available datasets used in this study are mentioned in this article and Supplementary Material. Data generated in this study are available under ArrayExpress E-MTAB-10320 (WGS), E-MTAB-10330 (RNA-seq tumors) and E-MTAB-10331 (RNA-seq organ-oids).

## Supplementary Materials

The following supporting information can be downloaded at: https://www.mdpi.com/article/10.3390/cancers14092166/s1, Supplementary Methods. DNA preparation and whole genome sequencing. Figure S1. Retinal organoid differentiation. Figure S2. Analysis of pRB expression in edited H9 hESCs. Figure S3. Whole genome sequencing of cell lines. Figure S4. Presence of retinal progenitors. Figure S5. Immunofluorescent staining of ganglion, bipolar and Müller glia cells. Figure S6. Immunofluorescent staining for rod photoreceptors. Figure S7. Quantification of Ki67-positive cells based on microscopy images. Figure S8. Immunofluorescent staining for LM-opsin and S-opsin. Figure S9. Ki67 in horizontal cells, rod photoreceptors and Müller glia. Figure S10. Retinal differentiation of H9_RB1ex1. Figure S11. Retinal differentiation of H9_RB1ex1. Figure S12. Retinal differentiation of H9_RB1ex1.Figure S13. Gene expression analysis in *RB1*^het^ samples of experiment 30. Figure S14. Gene expression of E2F transcription factors and cell cycle marker genes. Figure S15. Heatmaps of DEGs determined by multiparametric analysis. Figure S16. Gene set enrichment analysis (GSEA). Figure S17. Comparison of gene expression between organoids and retina. Figure S18. Comparison of gene expression in *RB1*^wt^ to retinal organoids generated by Kaya et al. Figure S19. Top 5 GO terms in fetal retina versus retinoblastoma. Figure S20. Parainflammation gene expression signature in *RB1*^ko^ organoids at d152. Supplementary file 1. Macro for signal quantification using ImageJ (three channels: green/red/blue). Supplementary file 2. Varbank export. Supplementary file 3. Varbank export after filtering for new variants. Table S1. Antibodies. Table S2. Samples for RNA-seq. Table S3. Predicted off-targets for gRNA targeting *RB1* exon 3.
