# Supplementary material for: RB1-Negative Retinal Organoids Display Proliferation of Cone Photoreceptors and Loss of Retinal Differentiation
Source: Cancers (Basel). 2022 Apr 26;14(9):2166. doi: 10.3390/cancers14092166 (PMC9105736; doi:10.3390/cancers14092166)
Supplement: Supplementary file 1 [file cancers-14-02166-s001.zip › cancers-1655176-supplementary-proof - send to xml - 2.pdf]

cancers MDPI

Article

RB1-Negative Retinal Organoids Display Proliferation of Cone Photoreceptors and Loss of Retinal Differentiation

Deniz Kanber, Julia Woestefeld, Hannah Döpper, Morgane Bozet, Alexandra Brenzel, Janine Altmüller, Fabian Kilpert, Dietmar Lohmann, Claudia Pommerenke and Laura Steenpass

Supplementary Methods: DNA preparation and whole genome sequencing

Genomic DNA was isolated using FlexiGene DNA Kit (QIAGEN) according to manual. Whole genome sequencing (150bp, paired-end, NovaSeq 6000) and post-processing using Varbank 2.0 (https://varbank.ccg.uni-koeln.de/varbank2/) was conducted by the Cologne Center for Genomics (CCG), University of Cologne. Varbank conducted read-alignment (BWA-mem), mapping to genome version GRCh38 with annotations based on Ensembl b90 running a GATK-style pipeline (RRID:SCR_001876). Variant calling used Samtools mpileup, GATK HaplotypeCaller and Plapytus for increased sensitivity. Settings were 'Datasets/Filtering' -> 'QC SNVs+short INDELs' -> 'Quorum level SNV'=1, resulting in 1,174 variants detected by at least one caller (Supplementary file 2). Filtering for new variants in homozygous or heterozygous cell lines was conducted ('Variants' -> 'A:PRID3850:SID106175:AID100177:75-H9' !~ '=') leaving 76 variants (Supplementary file 3). Removing variants that were duplicates on genomic level 58 variants that are private to or shared between *RB1*^{het} and *RB1*^{ko} remained (Fig. S1). Using Chopchop (RRID:SCR_015723; https://chopchop.cbu.uib.no/) with gRNA sequence TACAGAAAAACATAGAAATCAGG verified the specific mismatch 0 (MM0) binding within the *RB1* gene and estimated one MM2, and 46 MM3 potential off-targets (Table S3), whereby none of those overlapped (bedtools, RRID:SCR_006646) with the 76 new variants.

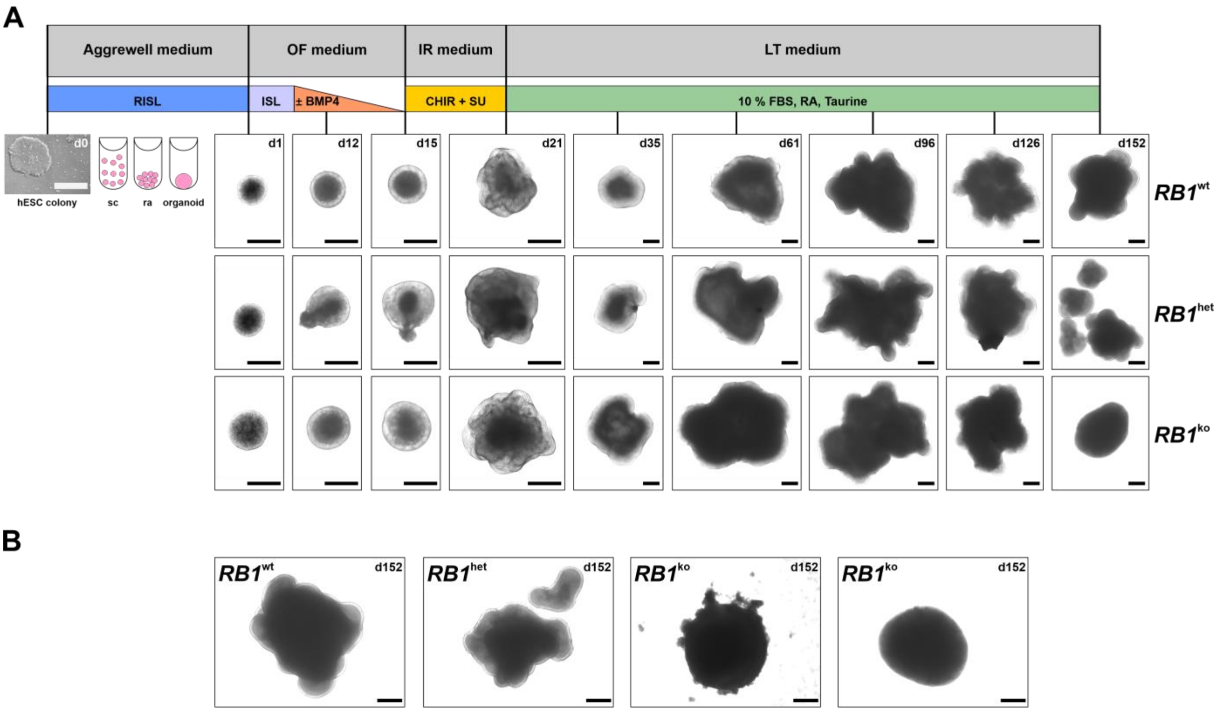

Figure S1. Retinal organoid differentiation. A) Differentiation is induced by single-cell aggregation and guided by small molecule addition. IR medium is supposed to enhance development of retinal pigment epithelium. Detailed protocol is described in [31]. Retinal tissue develops as bright outer rim from d12 onward. Genotype has no influence on morphology and size up to d126. Scale bar 50 µm. OF: organoid-forming medium, IR: induction reversal, LT: long term, R: ROCK inhibitor Y-27632, I: IWR1-endo, S: SB-431542, L: LDN-193189, CHIR: CHIR99021, SU: SU5402, RA: retinoic acid, FBS: fetal bovine serum, sc: single cell, ra: re-aggregation. B) Retinal organoids at d152. Genotypes *RB1*^{wt} and *RB1*^{het} show presence of bright outer photoreceptor layer and retinal loops, both structures get lost in *RB1*^{ko} between d126 and d152.

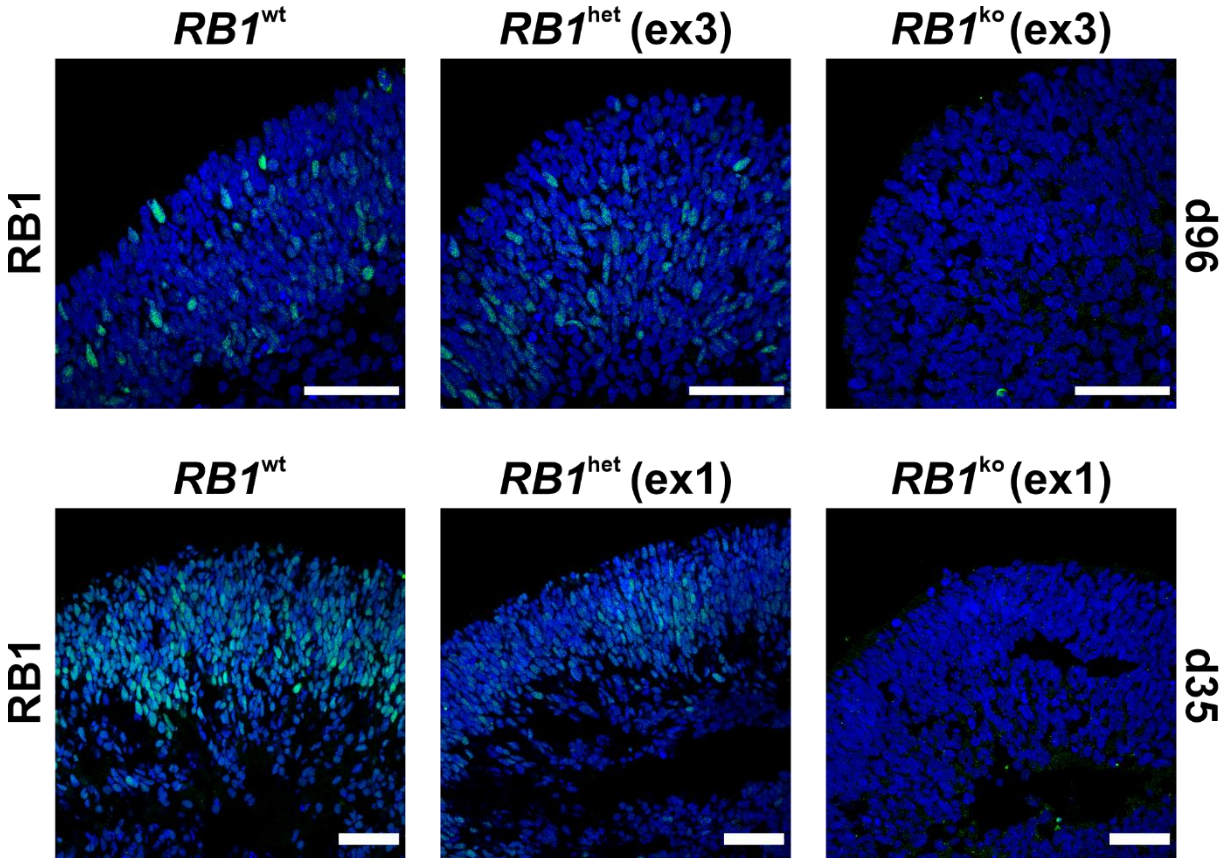

Figure S2. Analysis of pRB expression in edited H9 hESCs. Immunofluorescent staining for pRB (green) in retinal organoids differentiated from H9, H9_RB1ex3 (top) and H9_RB1ex1 cell lines (bottom). Nuclei were counterstained with DAPI (blue). Scale bar 50µm.

A

Sample	Sample ID	genotype	total reads	mean cov.	cov. 20x	variants
75-H9	100177	parental, wt	986,775,098	42	90.8	1096
76-C07	100178	ko	1,002,088,948	43	90.8	1088
77-G12LS	100179	het	970,577,054	41	90.7	1079

B

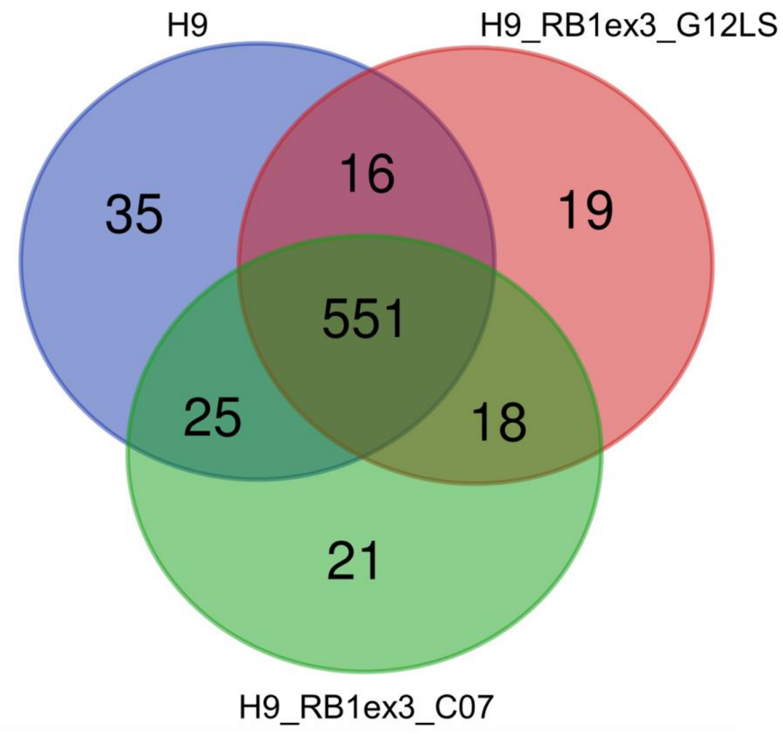

Figure S3. Whole genome sequencing of cell lines. **A**) Excerpt of the various QC numbers from Varbank. The full table from Varbank is available in Supplementary file 1. Cov.: coverage. **B**) Venn diagram of genomic variants detected in *RB1* genome-edited cell lines. Both edited cell lines carry a deletion of seven basepairs in exon 3 of *RB1*. H9_RB1ex3_G12LS in heterozygous, H9_RB1ex3_C07 in homozygous state [29]. No off-target sites were identified among variants not present in $RB1^{wt}$ (Table S3).

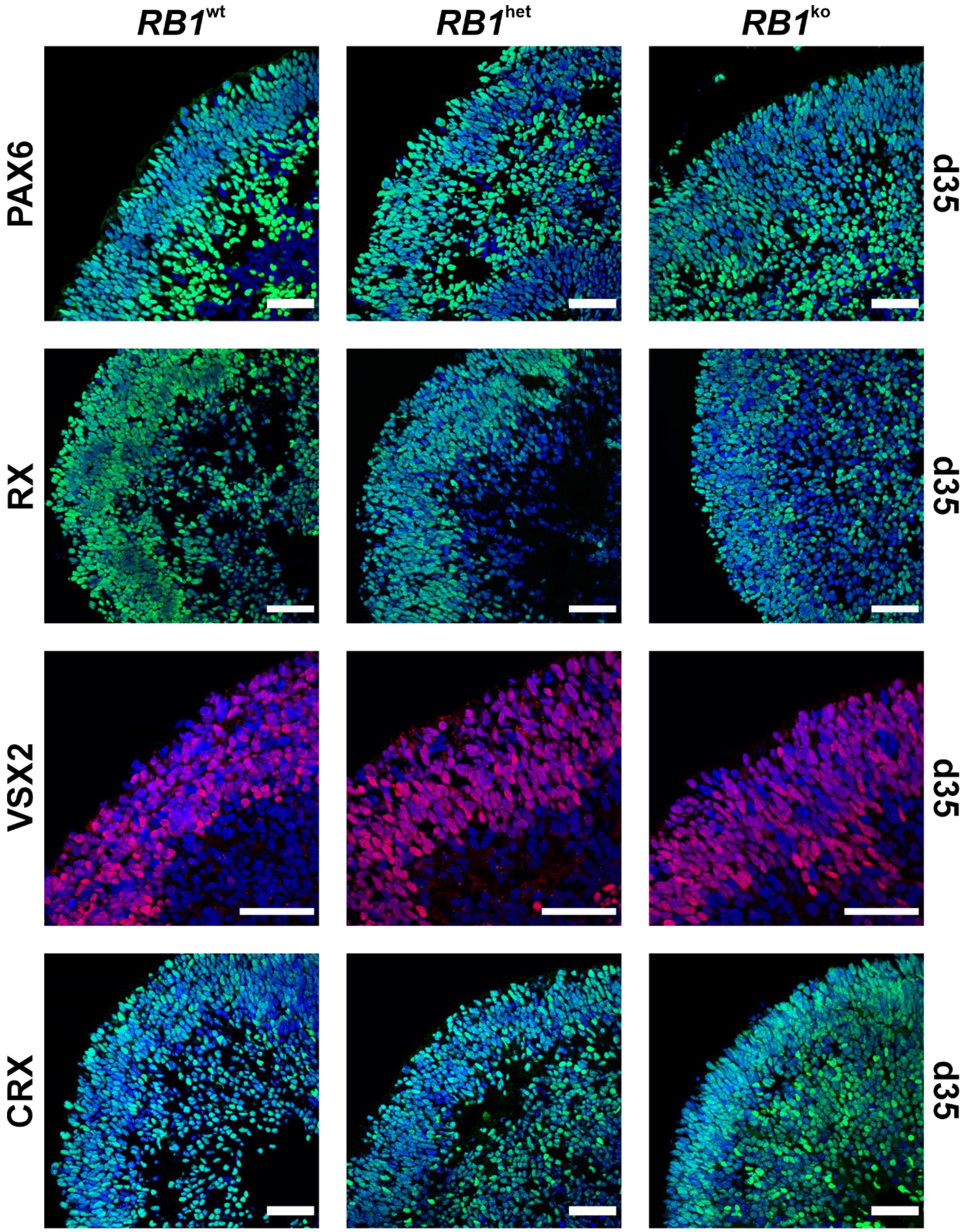

Figure S4. Presence of retinal progenitors. Representative images of retinal progenitors at d35 of differentiation stained for marker proteins PAX6 (green), RX (green), VSX2 (red) and CRX (green). Organoids of all three genotypes were organized into two layers, a dense outer layer with longitudinal cells and a loose inner layer. Progenitors are present in both structures. Nuclei were counterstained with DAPI (blue). Scale bar 50 μm.

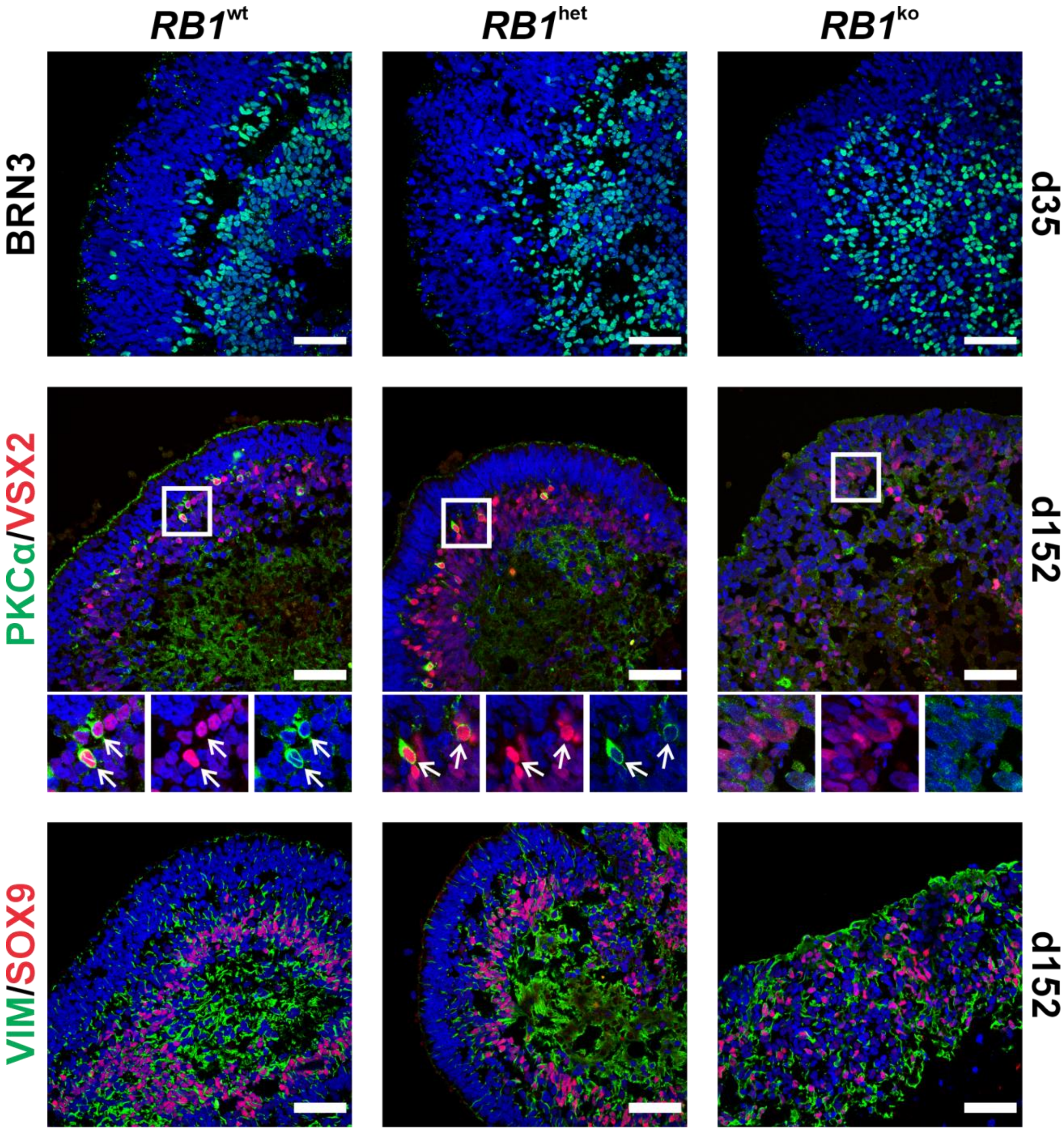

Figure S5. Immunofluorescent staining of ganglion, bipolar and Müller glia cells. Top: Ganglion cells (BRN3, green) at d35. Ganglion cells develop first and disappear later, probably to poor metabolic support and lack of neuronal input. Middle: Bipolar cells (PKCα/VSX2; green/red) at d152. Bottom: Müller glia (VIM/SOX9; green/red) at d152. Bipolar cells and Müller glia develop last. To accurately identify bipolar cells and Müller glia, staining for two marker proteins were used. Nuclei were counterstained with DAPI (blue), scale bar 50 µm. Representative images are shown.

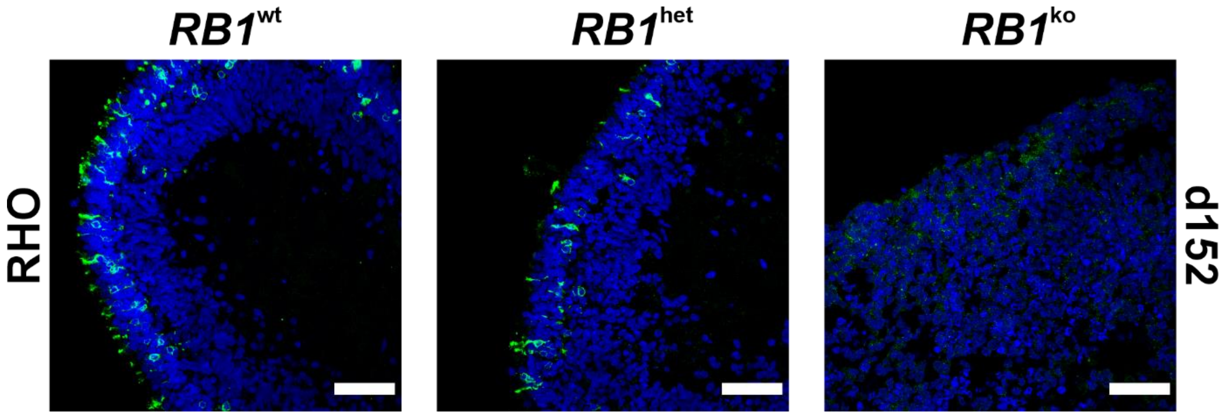

Figure S6. Immunofluorescent staining for rod photoreceptors. Representative images of mature rod photoreceptors (RHO, green) which were absent in *RB1*^{ko} organoids at d152. Nuclei were counterstained with DAPI (blue). Scale bar 50 μm.

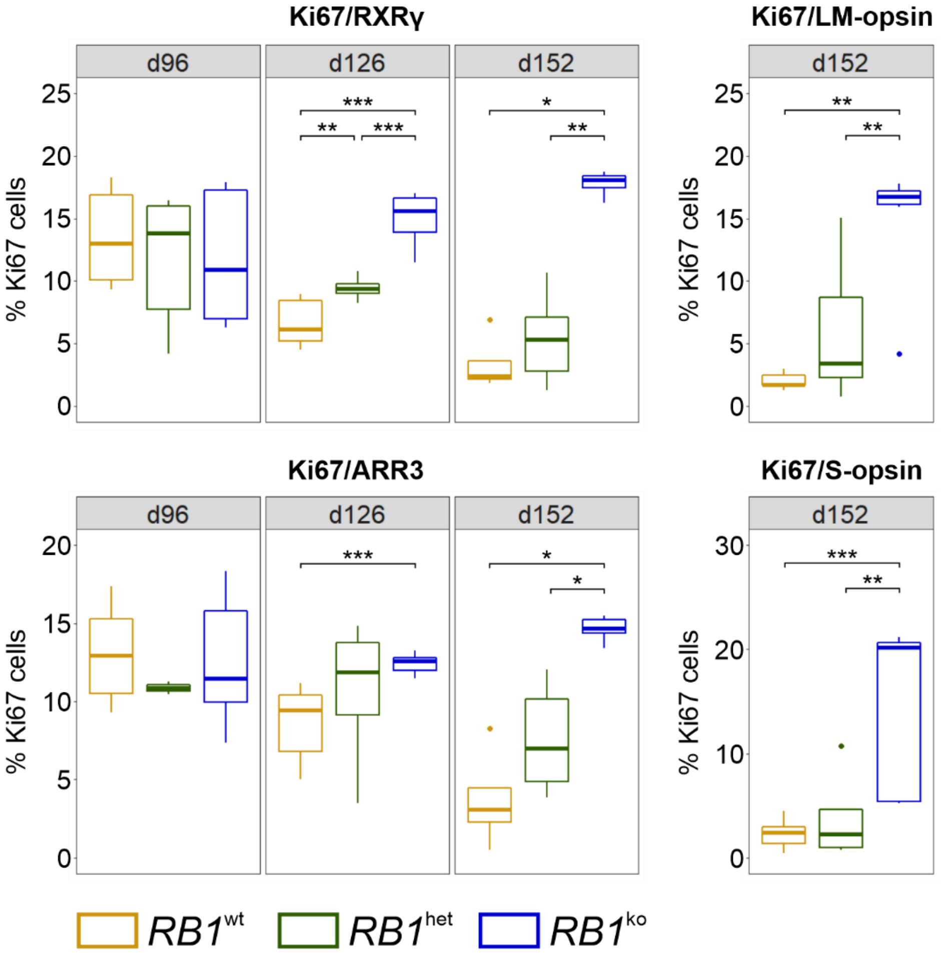

Figure S7. Quantification of Ki67-positive cells based on microscopy images. Data were determined in separate co-staining experiments for the indicated marker proteins. Percentages of Ki67-positive cells per total DAPI positive area are given. *p≤0.05, **p≤0.01, ***p≤0.001.

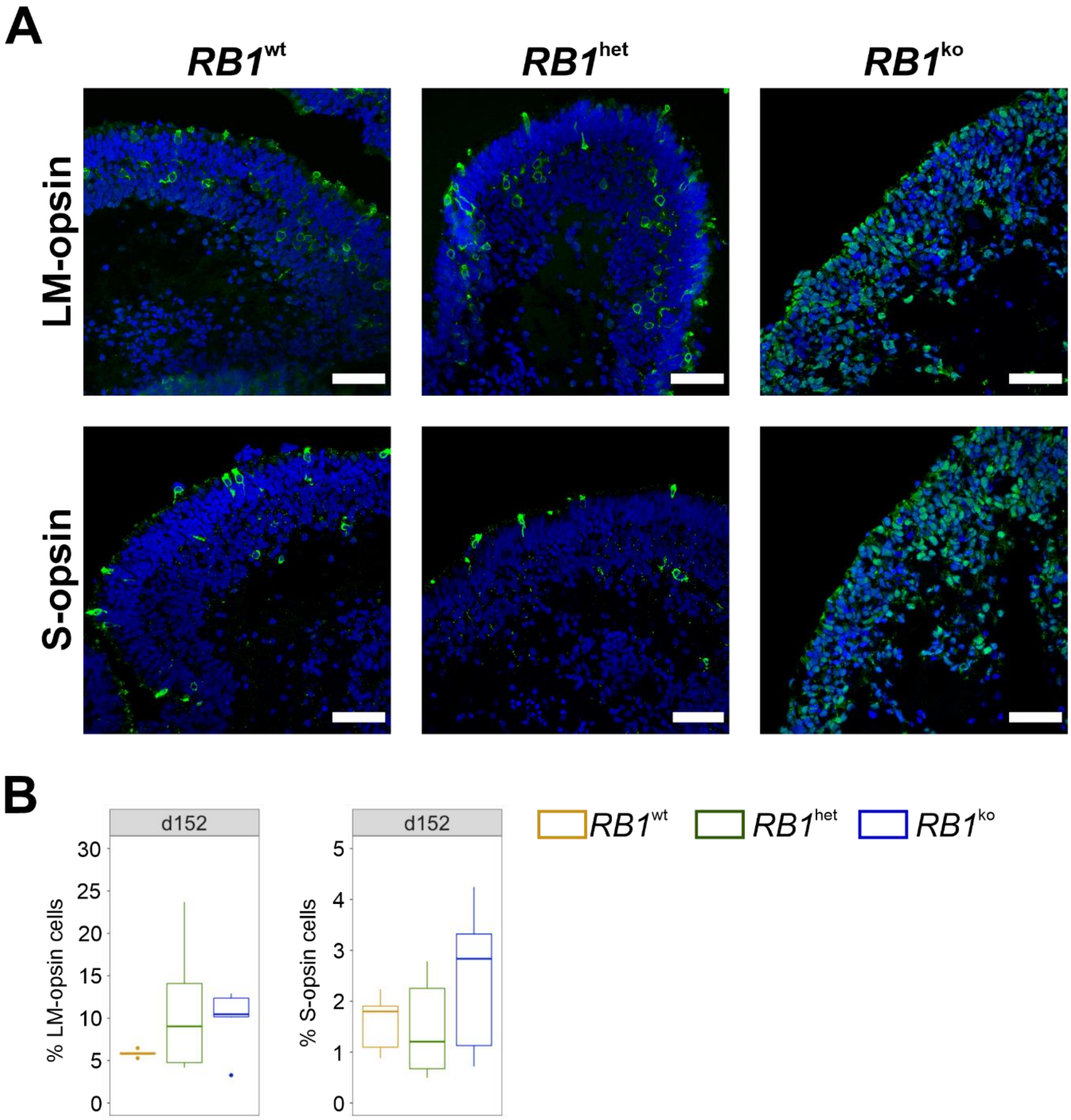

Figure S8. Immunofluorescent staining for LM-opsin and S-opsin. **A**) Representative images of LM-opsin and S-opsin (green) positive cone photoreceptor cells. Nuclei were counterstained with DAPI (blue). Scale bar 50 µm. **B**) Quantification of LM-opsin and S-opsin positive cone photoreceptor cells based on microscopy images. Percentages of marker-positive cells per total DAPI positive area are given. Results were not significant.

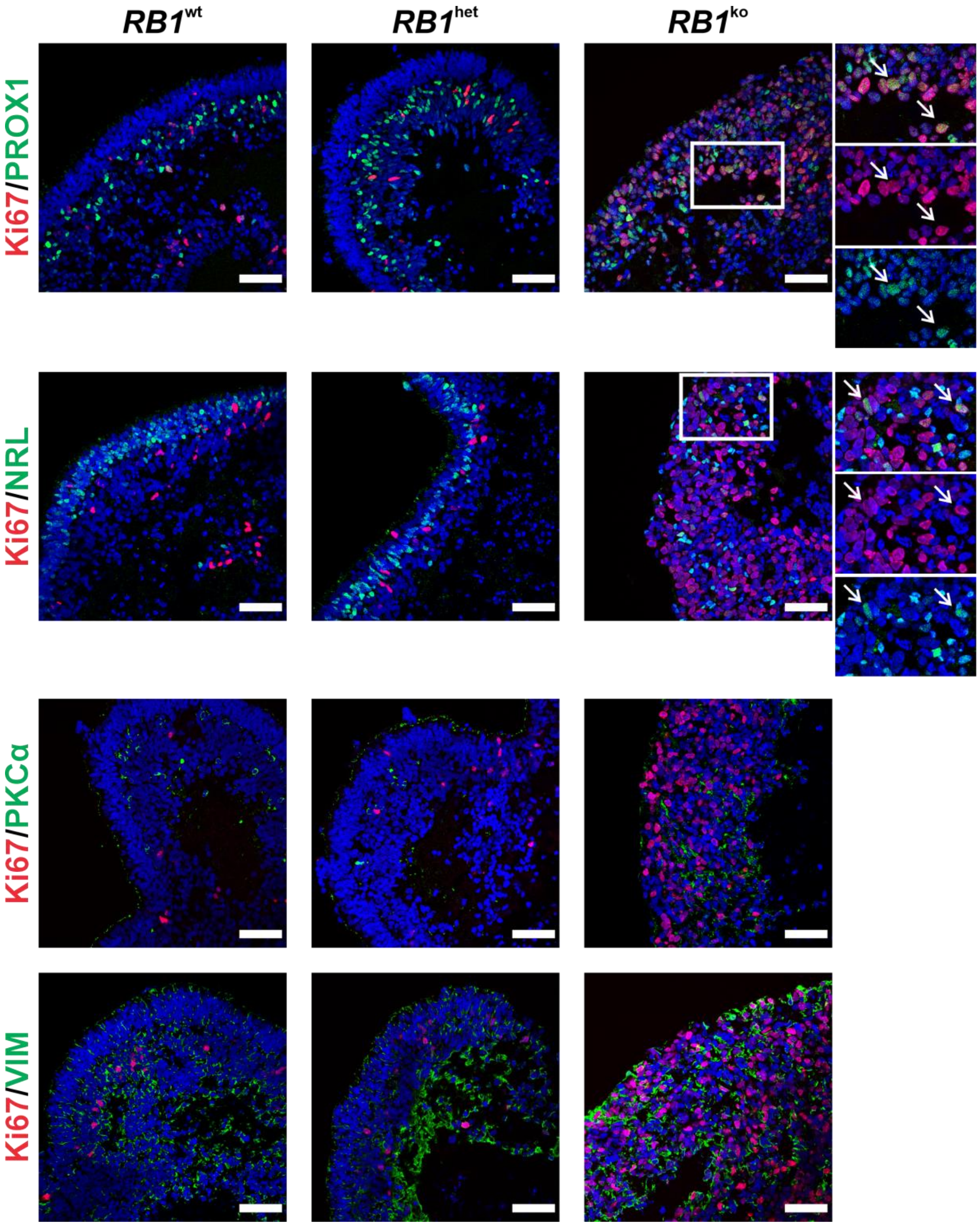

Figure S9. Ki67 in horizontal cells, rod photoreceptors and Müller glia. Representative images of co-immunofluorescent staining (d152) of cell-type-specific marker proteins (green) with proliferative marker Ki67 (red) for horizontal cells (PROX1), rod photoreceptors (NRL), bipolar cells (PKCα) and Müller glia cells (VIM). Arrows indicate double-positive cells. Scale bar is 50 μm. Nuclei were counterstained with DAPI (blue). Horizontal cells and rod photoreceptors were reduced in *RB1*^{ko} organoids, bipolar cells were absent, and appearance of Müller glia was comparable to *RB1*^{wt} and *RB1*^{het} organoids. Ki67 positive cells were more abundant in *RB1*^{ko} organoids and in these, layered structure observed in *RB1*^{wt} and *RB1*^{het} organoids was lost.

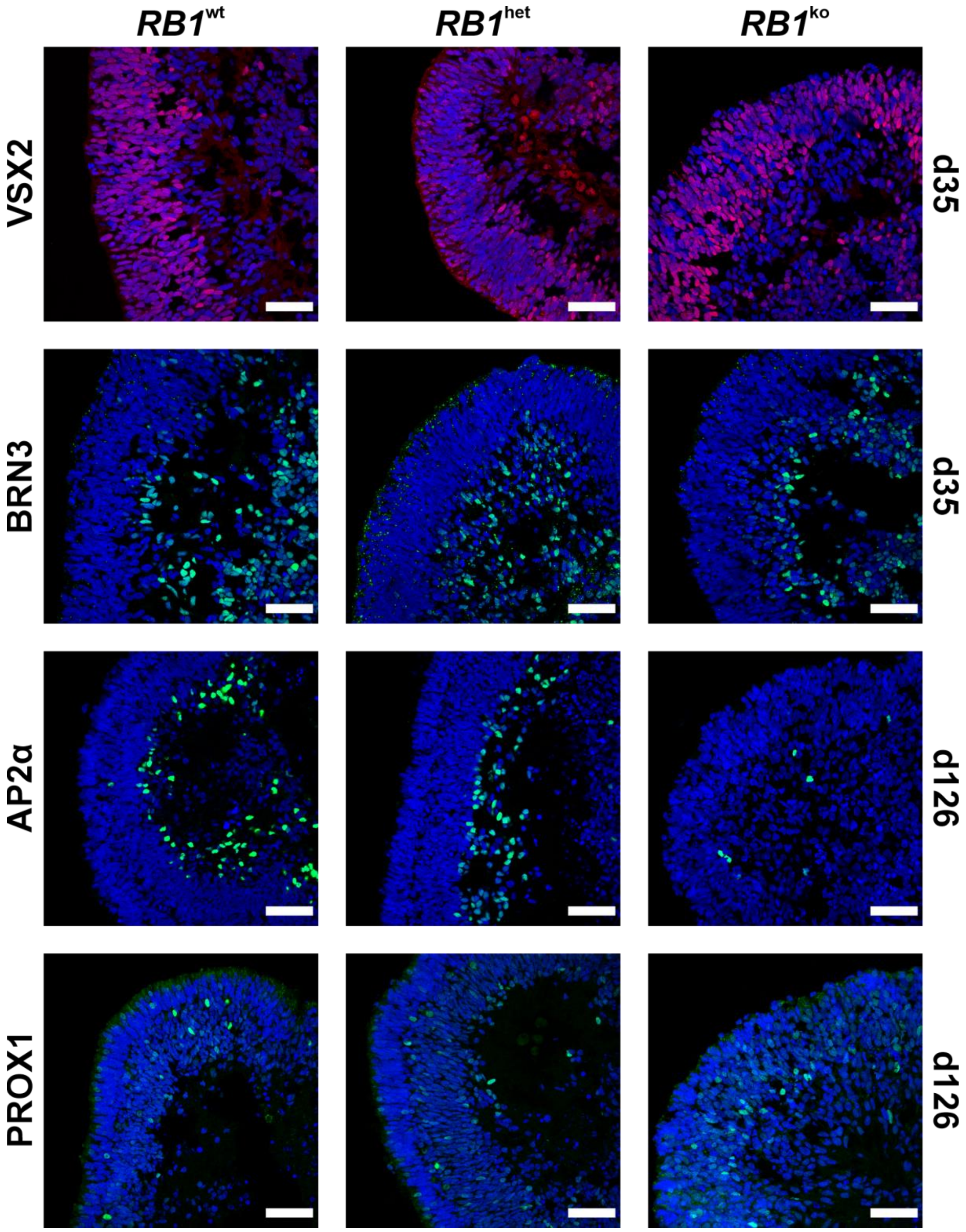

Figure S10. Retinal Differentiation of H9_RB1ex1. Representative images of immunofluorescent staining of retinal organoids differentiated from H9_RB1ex1_8B3 (het) and H9_RB1ex1_8F2 (ko) cell lines as described. Marker proteins BRN3, VSX2, AP2α and PROX1 were stained in green or red, nuclei were counterstained with DAPI (blue). Scale bar 50 μm.

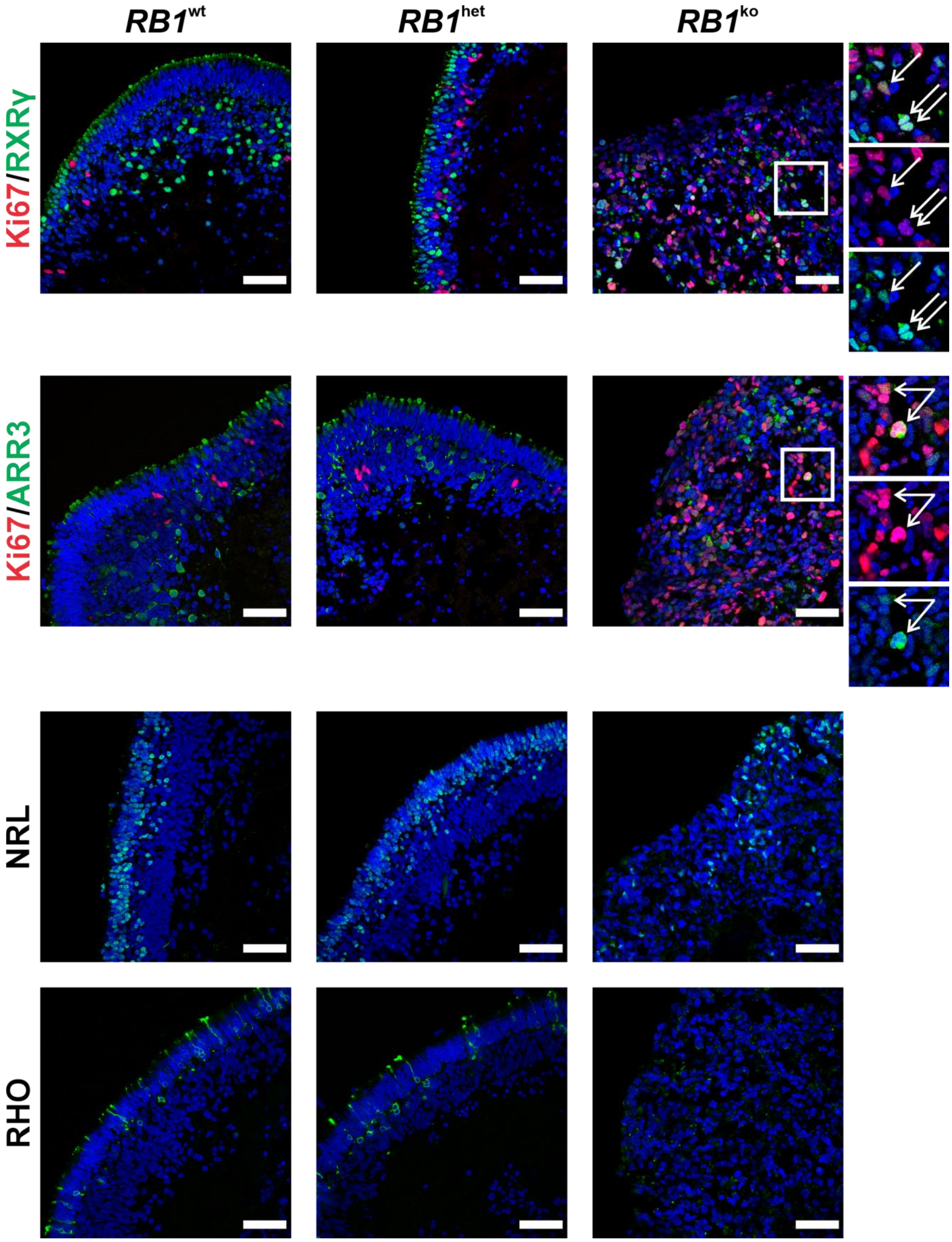

Figure S11. Retinal Differentiation of H9_RB1ex1. Representative images of immunofluorescent staining (d152) for cone photoreceptors (RXRγ, ARR3) and rod photoreceptors (NRL, RHO) of retinal organoids differentiated from H9_RB1ex1_8B3 (het) and H9_RB1ex1_8F2 (ko) cell lines as described. Marker proteins were stained in green, proliferation marker Ki67 in red, nuclei were counterstained with DAPI (blue). Area in the rectangle is enlarged; arrows indicate double-positive cells. Scale bar 50 µm.

.

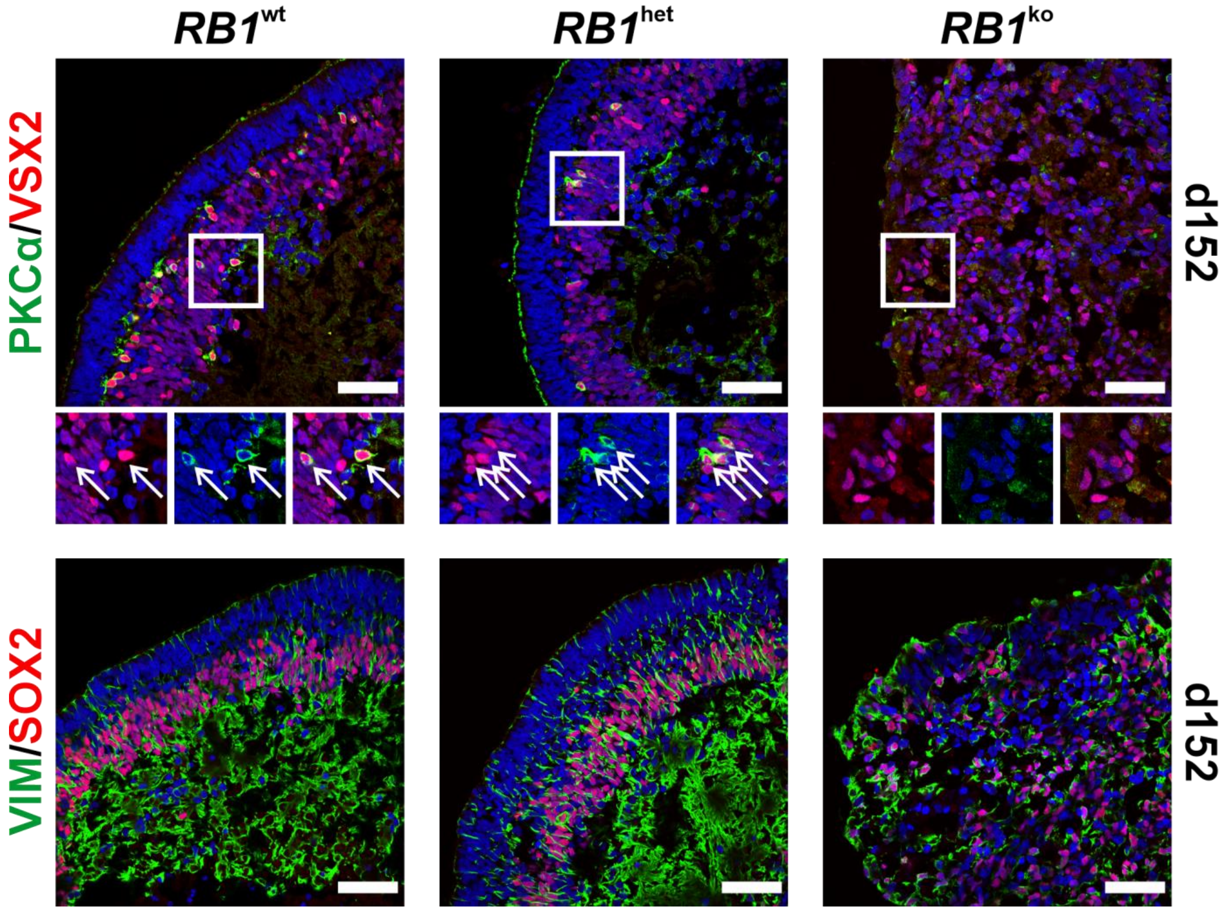

Figure S12. Retinal Differentiation of H9_RB1ex1. Representative images of immunofluorescent staining for bipolar (PKCα/VSX2, green/red) and Müller glia cells (VIM/SOX2, green/red) of retinal organoids differentiated from H9_RB1ex1_8B3 (het) and H9_RB1ex1_8F2 (ko) cell lines as described. Area in the rectangle is enlarged; arrows indicate double-positive cells. Nuclei were counterstained with DAPI (blue). Scale bar 50 μm.

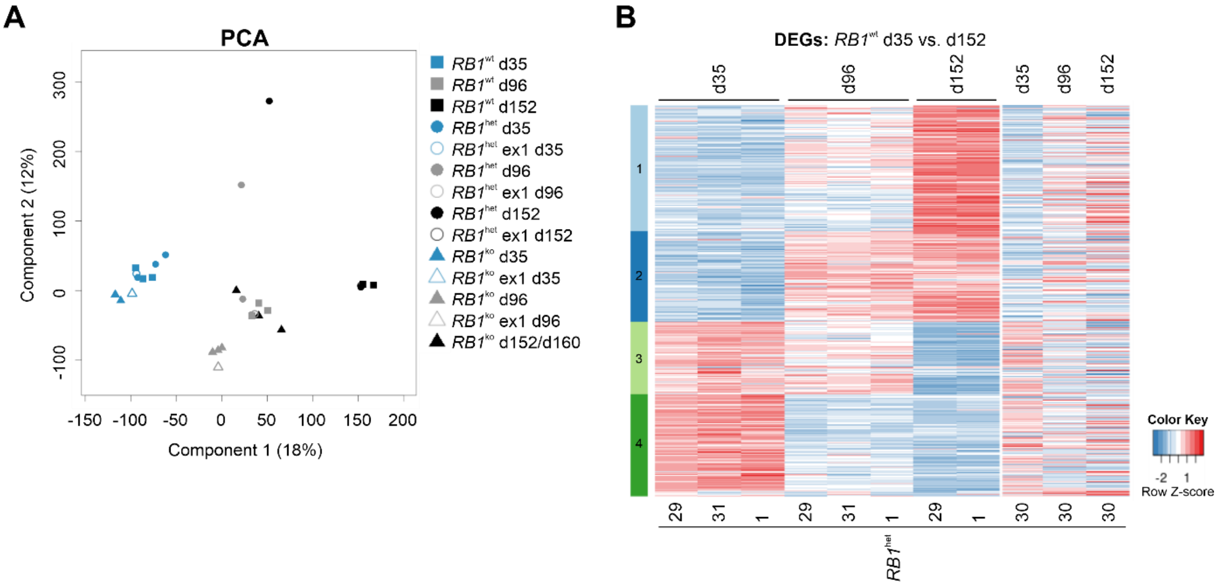

Figure S13. Gene expression analysis in *RB1*het samples of experiment 30. A) PCA of RNA-seq analyses including all samples. Samples *RB1*het are depicted as circles, identifying two samples as outliers (d96 and d152). Look up of sample indexes showed that both samples belong to experiment 30. B) DEGs and clusters are as shown in Figure 5. Number of differentiation experiment as specified in Table S2 is given below the plot. *RB1*het samples showed same expression patterns as *RB1*wt samples, except for samples of experiment 30. Samples of this experiment were therefore excluded from further analyses. p-adjust<0.05, log2(FC)>1.

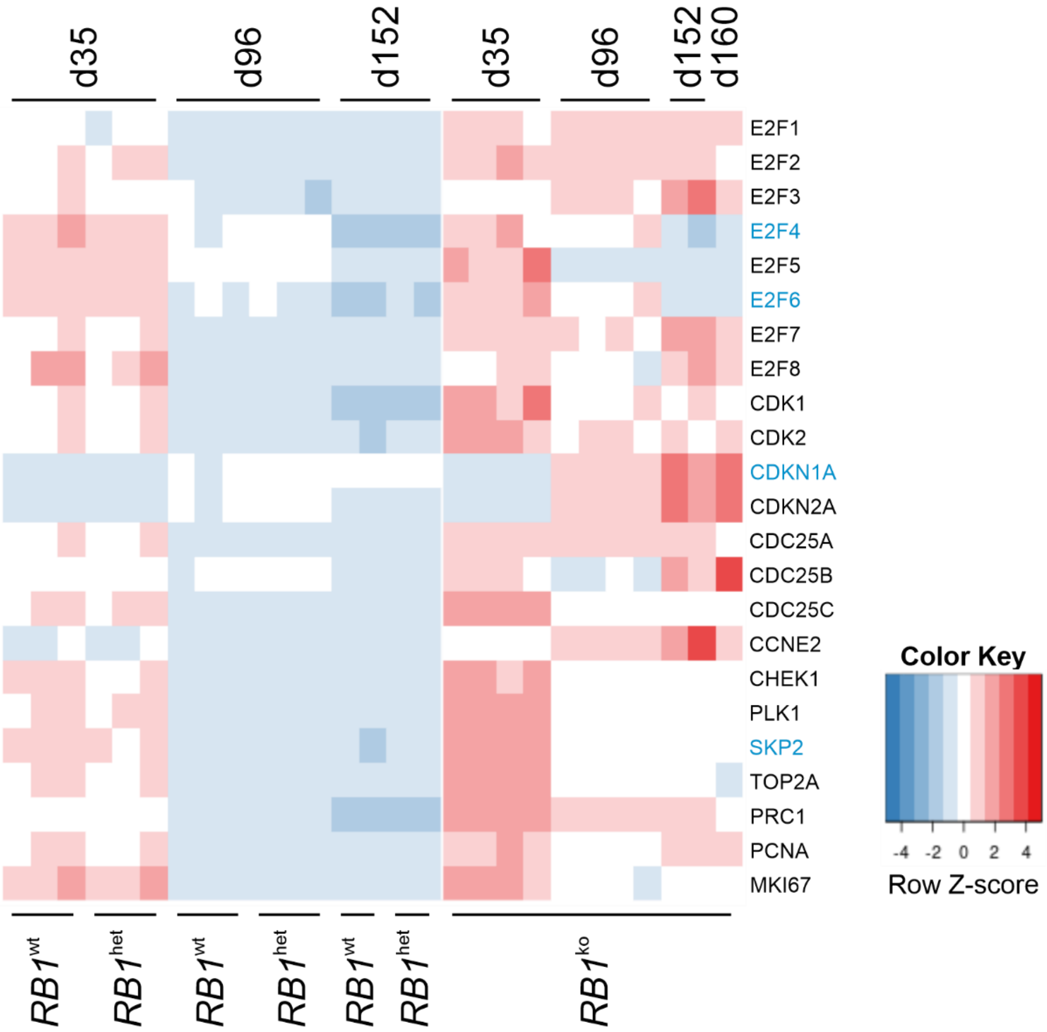

Figure S14. Gene expression of E2F transcription factors and cell cycle marker genes. Genes identified as DEG in the multiparametric analysis of Figure 6 (p-adjust<0.05, log2(FC)>1) are written in black, genes written in blue do not show significant differential gene expression between *RB1*^{wt}/*RB1*^{het} and *RB1*^{ko} across the differentiation time course.

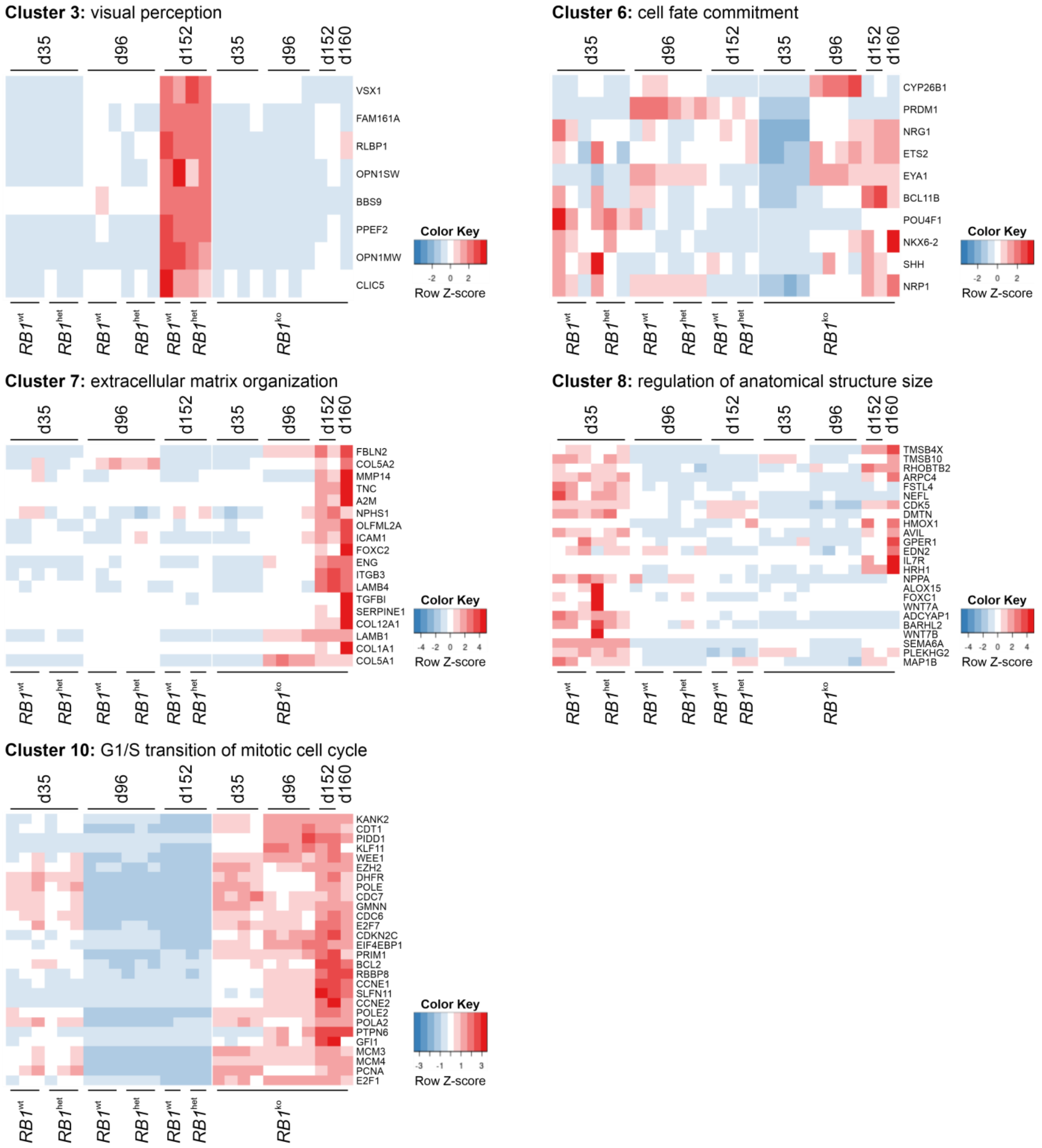

Figure S15. Heatmaps of DEGs determined by multiparametric analysis. Multiparametric analysis by DESeq2 (p-adjust<0.05, log2(FC)>1) considered 2 genotypes, three time points, and batches of experiments. Top GO terms of clusters showing differential gene expression profiles (Figure 6) are depicted here in detail.

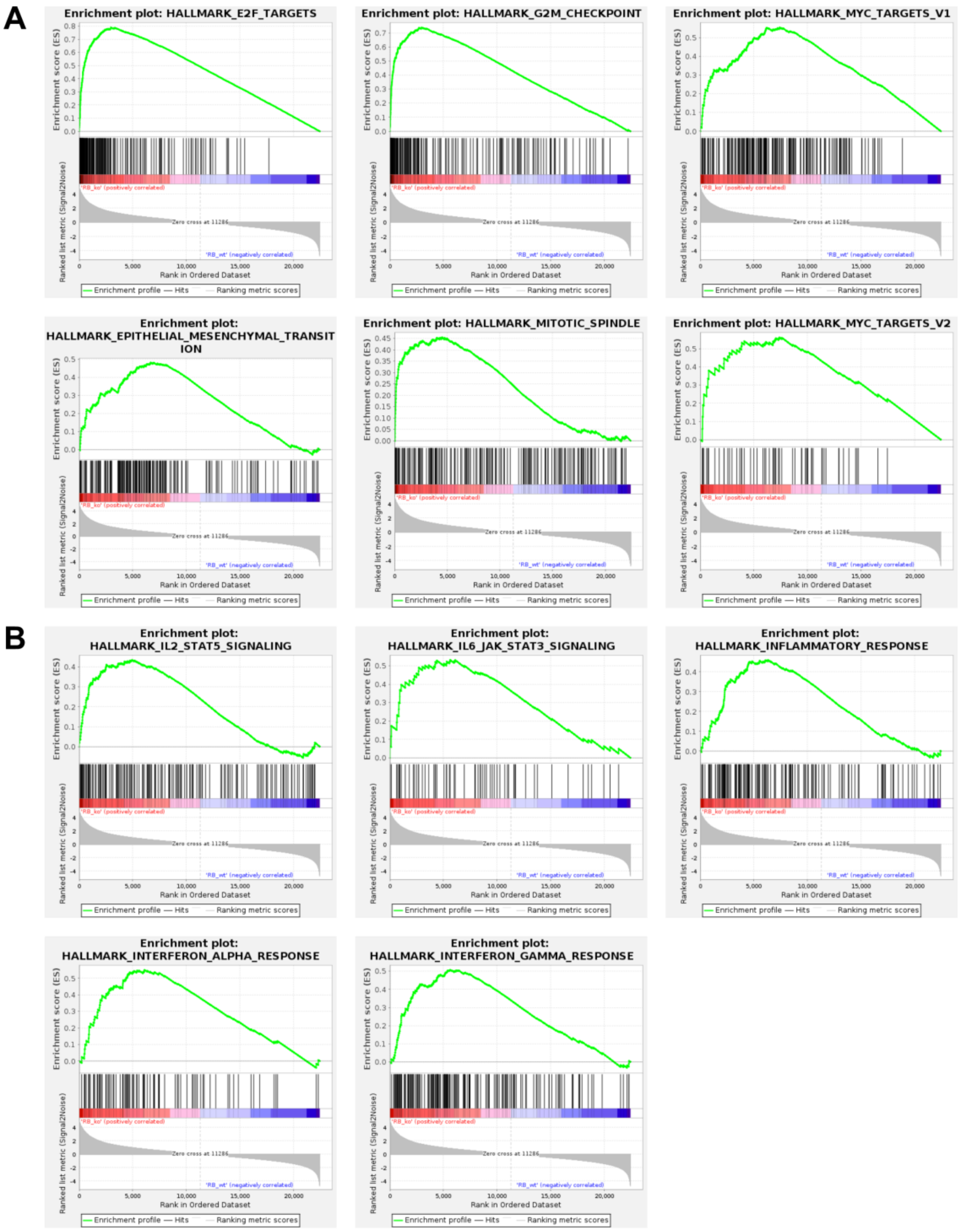

C

gene set name	ES	NES	FDR q-val
E2F_TARGETS	0.790	3.387	0
G2M_CHECKPOINT	0.739	3.154	0
MYC_TARGETS_V1	0.553	2.378	0
INTERFERON_GAMMA_RESPONSE	0.507	2.124	0
INTERFERON_ALPHA_RESPONSE	0.551	2.121	0
EPITHELIAL_MESENCHYMAL_TRANSITION	0.482	2.057	0
MYC_TARGETS_V2	0.561	1.999	0
MITOTIC_SPINDLE	0.455	1.964	0.000125
IL6_JAK_STAT3_SIGNALING	0.532	1.944	0.000102
INFLAMMATORY_RESPONSE	0.460	1.911	0.000273
IL2_STAT5_SIGNALING	0.436	1.844	0.000408

Figure S16. Gene set enrichment analysis (GSEA). Enrichment of genes in hallmark gene sets of MSigDB was analyzed using normalized count matrix of samples at d152. A) Plots for proliferation associated gene sets. B) Plots for immune associated gene sets. C) Table of GSEA results. ES: enrichment score, NES: normalized enrichment score, FDR q-val: false discovery rate q-value.

.

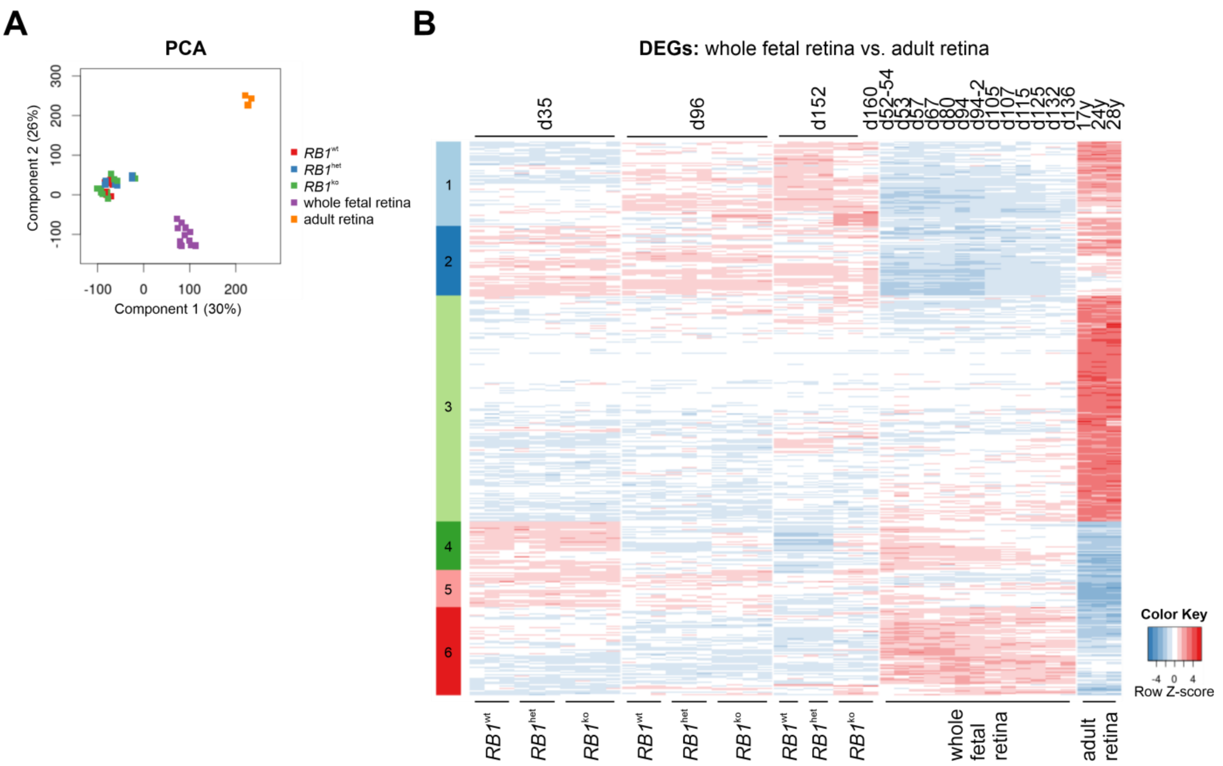

Figure S17. Comparison of gene expression between organoids and retina. A) Principal component analysis. Organoids were more similar to fetal than to adult retina. B) DEGs between fetal and adult retina were determined (p<0.001, log2(FC)>2) and expression of these genes in organoids was compared. Top enriched GO-terms of clusters were: c1: sensory perception of light stimulus; c2: none; c3: cilium movement; c4: nuclear division; c5: none; c6: positive regulation of neurogenesis. Expression in clusters c1, c4 and c5 is similar to fetal retina at d35 but to adult retina at d152, mimicking *in vivo* retina development. Samples were sorted first to time than to genotype. Following publicly available datasets were used: fetal retina: GSE104827, adult retina: NEI commons (dataset Retina Aging, samples 17yo.Retina.Norm, 24yo.Retina.Norm, 28yo.Retina.Norm).

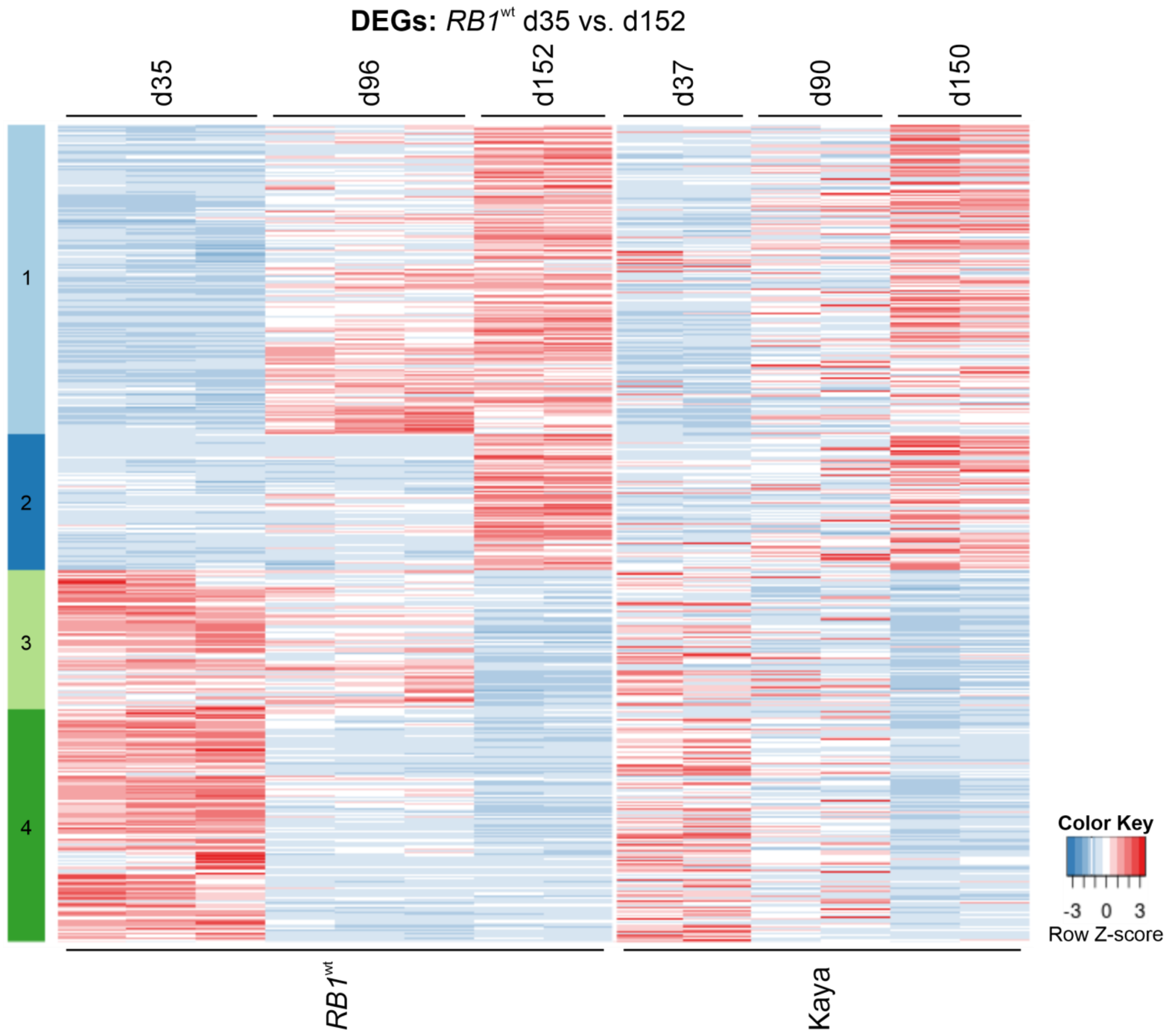

Figure S18. Comparison of gene expression in *RB1*wt to retinal organoids generated by Kaya et al. Expression of DEGs determined in *RB1*wt (d35 versus d152, Figure 5B) was compared to retinal organoids of H9 hESCs generated and analyzed by Kaya et al.(dataset GSE129104) [39]. Patterns were similar, especially for comparison of d35 vs Kaya d37 and d152 vs Kaya d150. Kaya et al. used a differentiation protocol relying on generation and plating of embryoid bodies and subsequent transfer of optic structures into suspension. It is unclear if single or pools of organoids were used for transcriptome analyses. Use of single organoids most likely results in more variation in gene expression. p-adjust<0.05, log2(FC)>1.

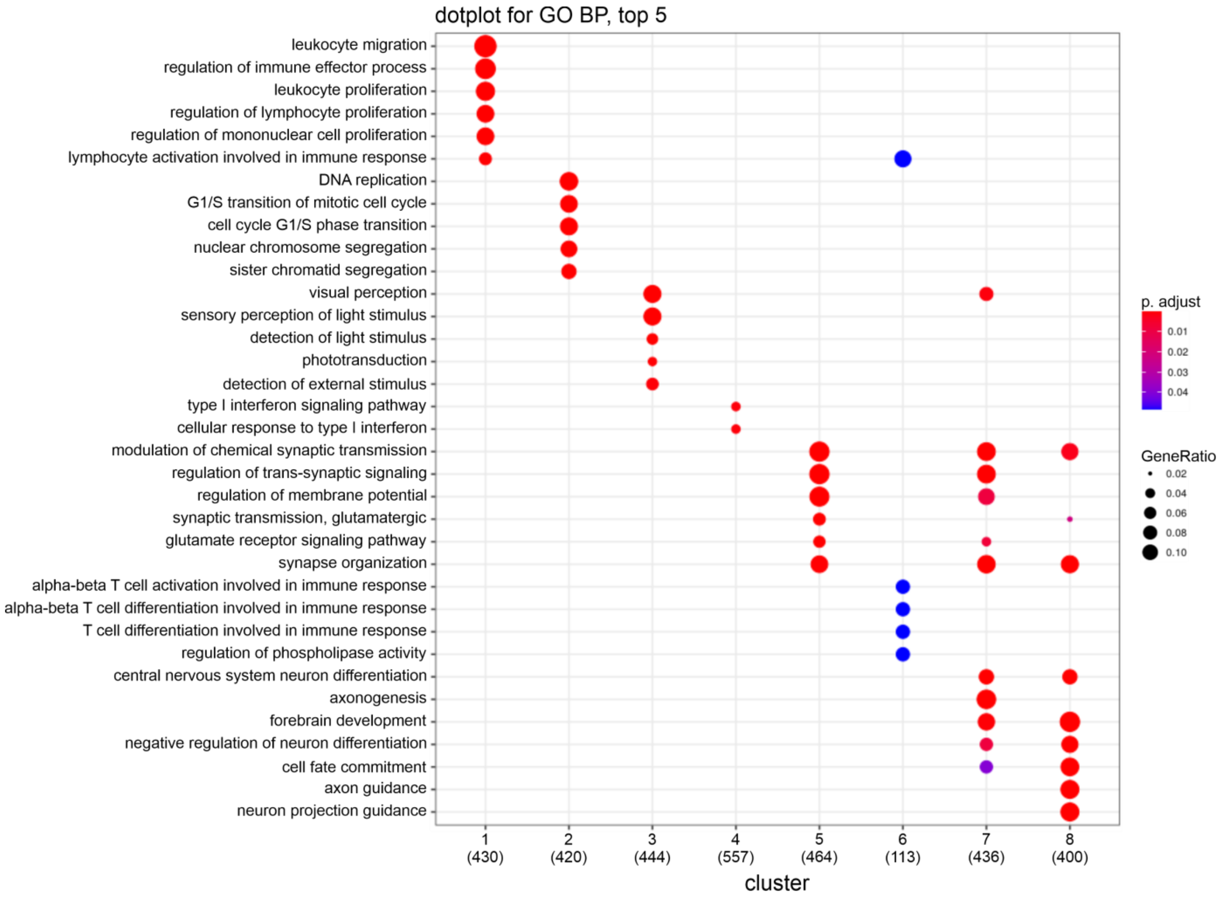

Figure S19. Top 5 GO terms in fetal retina versus retinoblastoma. DEGs between fetal retina and five retinoblastoma samples were determined and subjected to cluster analysis (Figure 7B). Top 5 (p-adjust<0.05) enriched biological processes per cluster determined by reduced redundancy GO analysis. The total number of DEGs in each cluster associated with a GO-term is given in parentheses, dot size: ratio of number of DEGs present in the specific GO-term versus total number GO-term associated DEGs in this cluster, dot color: p-adjust. If any of the top 5 terms of one cluster is present in another cluster, corresponding dots are plotted.

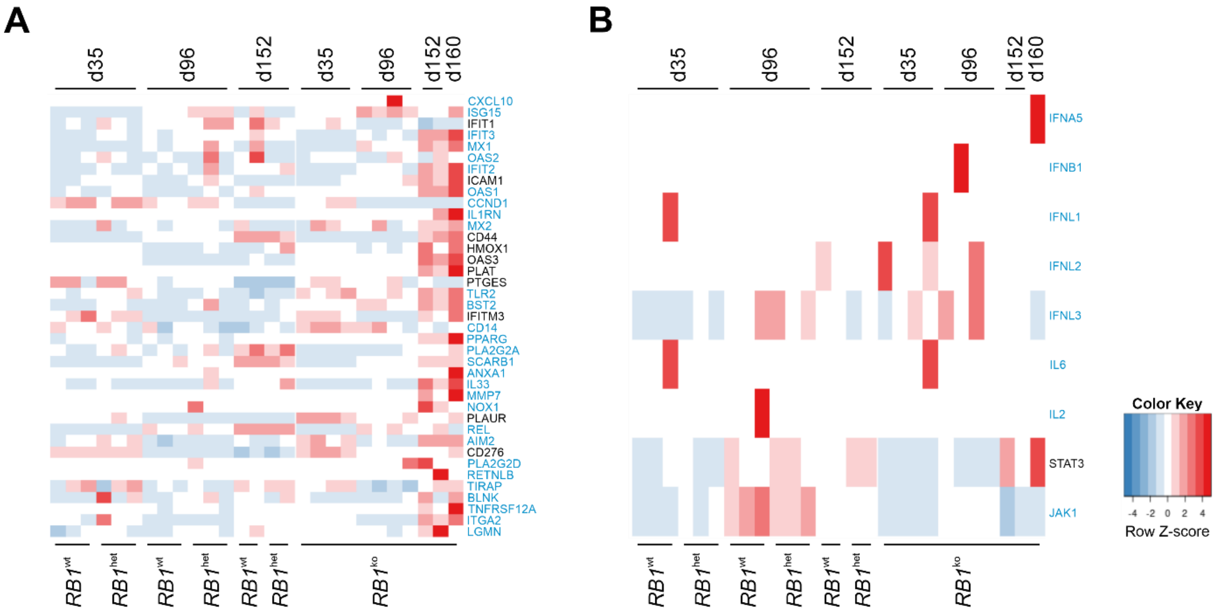

Figure S20. Parainflammation gene expression signature in *RB1*^{ko} organoids at d152. A) Heatmap depicts expression of genes defined as parainflammation signature [49]. CXCL9 is not included since it showed no reads in our data set. B) Expression heatmap of genes encoding secreted immune regulators and related genes. *IFN* genes not listed had no reads. Genes identified as DEG in the multiparametric analysis of Figure 6 (p-adjust<0.05, log2(FC)>1) are written in black, genes written in blue do not show significant differential gene expression between *RB1*^{wt}/*RB1*^{het} and *RB1*^{ko} across the differentiation time course.

Supplementary file 1. Macro for signal quantification using ImageJ (three channels: green/red/blue).

Supplementary file 2. Varbank export. The file DK01.variants_all.xlsx contains the export after applying the 'Datasets/Filtering' -> 'QC SNVs+short INDELs' -> 'Quorum level SNV'=1 setting to the Varbank default. The Table comprises 1,174 variants that were detected by at least on variant caller. The 'MedPred'-value is the median of all pathogenicity rank scores obtained from the dbNSFP database.

Supplementary file 3. Varbank export after filtering for new variants. The file DK01.variants_filtered.xlsx contains the result of applying Varbank setting ('Variants' -> 'A:PRID3850:SID106175:AID100177:75-H9' !~ '=') to Supplementary file 1. The 'MedPred'-value is the median of all pathogenicity rank scores obtained from the dbNSFP database.

Table S1. Antibodies.

Antibody	Company	Order no	Dilution	RRID
**Primary antibodies**				
Mouse anti-human retinoblastoma protein clone G3-245	BD Pharmingen	554136	1:200	RRID:AB_395589
Mouse anti-Ki-67 clone B56	BD Pharmingen	550609	1:200	RRID:AB_393778
Mouse anti Retinal RX (G-12)	Santa Cruz	sc-271889	1:200	RRID:AB_10708730
Sheep anti-human Chx10 (VSX2) (CT)	Exalpha	X1179P	1:300	RRID:AB_2999828
Mouse anti-human CRX (M02) clone 4G11	Abnova	H00001406-M02	1:1000	RRID:AB_425381

Goat anti-BRN3 clone C13	Santa Cruz	sc-6026	1:100	RRID:AB_673441
Rabbit anti-PROX1	Millipore	AB5475	1:2000	RRID:AB_177485
Mouse anti-AP2	DSHB	3B5-s	2mg/ml	
Rabbit anti-RXR	SpringBio	E4332	1:500	RRID:AB_11218714
Rabbit anti-ARR3	LifeSpan Biosciences	LS-C382134-100	1:250	
Goat anti-NRL	R&D Biosystems	AF2945	1:100	RRID:AB_2155098
Mouse anti-RHO clone RET-P1	Sigma-Aldrich	MAB5316	1:50	RRID:AB_2156055
Mouse anti-PRKC clone H-7	Santa Cruz	sc-8393	1:100	RRID:AB_628142
Mouse anti-VIM clone E-5	Santa Cruz	sc-373717	1:100	RRID:AB_10917747
Rabbit anti-SOX9 clone D8G8H	Cell Signaling	82630S	1:400	RRID:AB_2665492
Rabbit anti-opsin, blue (S-opsin)	Millipore	AB5407	1:300	RRID:AB_177457
Rabbit anti-opsin, red/green (LM-opsin)	Millipore	AB5405	1:300	RRID:AB_177456
**Secondary antibodies**				
Rabbit anti-Goat IgG (H+L), Alexa Fluor® 488	Thermo Fisher	A-11078	1:1000	RRID:AB_2534122
Donkey anti-Sheep IgG (H+L), Alexa Fluor® 555	Thermo Fisher	A-21436	1:1000	RRID:AB_2535857
Anti-mouse IgG (H+L), Alexa Fluor® 488	Cell Signaling	4408S	1:1000	RRID:AB_10694704
Anti-mouse IgG (H+L), Alexa Fluor® 555	Cell Signaling	4409S	1:1000	RRID:AB_1904022
Anti-rabbit IgG (H+L), Alexa Fluor® 488	Cell Signaling	4412S	1:1000	RRID:AB_1904025
Anti-rabbit IgG (H+L), Alexa Fluor® 555	Cell Signaling	4413S	1:1000	RRID:AB_10694110
Anti-mouse IgG, HRP	GEHealthcare	NA931V	1:10000	RRID:AB_772210

Table S2. Samples for RNA-seq. RNA of each sample was prepared from three organoids pooled after RNA isolation. a: samples 3 and 17 contained RNA form only one organoid, samples 8 and 19 contained RNA form two pooled organoids. b: samples excluded from RNA-Seq analysis based on PCA and cluster dendrogram analyses. Seventeen samples carried a 7 basepair deletion in exon 3 of *RB1* [29), five samples carried a complete deletion of *RB1* exon1 [30]. Day: day of differentiation, experiment: independent repetitions of retinal differentiation.

no	sample name	genotype	type of mutation	day	experiment
1	H9_d35_29DK	$RB1^{wt}$	-	35	29
2	H9_d35_1HD	$RB1^{wt}$	-	35	1
3	H9_d35_7JM[a]	$RB1^{wt}$	-	35	7
4	H9_d96_29DK	$RB1^{wt}$	-	96	29
5	H9_d96_1HD	$RB1^{wt}$	-	96	1
6	H9_d96_7JM	$RB1^{wt}$	-	96	7
7	H9_d152_29DK	$RB1^{wt}$	-	152	29
8	H9_d152_1HD[a]	$RB1^{wt}$	-	152	1
9	G12LS_d35_29DK	$RB1^{het}$	del7/ex3	35	29
10	G12LS_d35_30DK[b]	$RB1^{het}$	del7/ex3	35	30
11	G12LS_d35_31DK	$RB1^{het}$	del7/ex3	35	31
12	8B3_d35_1HD	$RB1^{het}$	deletion ex1	35	1
13	G12LS_d96_29DK	$RB1^{het}$	del7/ex3	96	29
14	G12LS_d96_30DK[b]	$RB1^{het}$	del7/ex3	96	30
15	G12LS_d96_31DK	$RB1^{het}$	del7/ex3	96	31
16	8B3_d96_1HD	$RB1^{het}$	deletion ex1	96	1
17	G12LS_d152_29DK[a]	$RB1^{het}$	del7/ex3	152	29
18	G12LS_d152_30DK[b]	$RB1^{het}$	del7/ex3	152	30
19	8B3_d152_1HD[a]	$RB1^{het}$	deletion ex1	152	1
20	C07_d35_29DK	$RB1^{ko}$	del7/ex3	35	29
21	C07_d35_30DK	$RB1^{ko}$	del7/ex3	35	30
22	C07_d35_31DK	$RB1^{ko}$	del7/ex3	35	31
23	8F2_d35_1HD	$RB1^{ko}$	deletion ex1	35	1
24	C07_d96_29DK	$RB1^{ko}$	del7/ex3	96	29
25	C07_d96_30DK	$RB1^{ko}$	del7/ex3	96	30
26	C07_d96_31DK	$RB1^{ko}$	del7/ex3	96	31
27	8F2_d96_1HD	$RB1^{ko}$	deletion ex1	96	1
28	C07_d152_29DK	$RB1^{ko}$	del7/ex3	152	29
29	C07_d152_30DK	$RB1^{ko}$	del7/ex3	152	30
30	C07_d160_7JM	$RB1^{ko}$	del7/ex3	160	7

Table S3. Predicted off-targets for gRNA targeting *RB1* exon 3. Prediction based on https://chopchop.cbu.uib.no/. Position: position of first nucleotide in hg38.

no	position	sequence	gene	location
1	chr1:16378014	TACAGAAAcACAgAGAAAgCAGG	SZRD1	intron 1
2	chr1:160557515	CCAcATTTCTgTGTTTTTCTGcA	CD84	intron 1
3	chr10:44709936	TACAaAAcAACATAGAAAcCAGG		intergenic
4	chr10:105719120	CCAGAgTTCTgTGTcTTTCTGTA	LINC02627	intron 3
5	chr12:15735675	TACAaAtAAACATAGtAATCAGG	EPS8	intron 4
6	chr12:49243439	TcCAGAAAAAaAaAGAAATCAGG	TUBA1C	intron 1
7	chr13:47519932	CCCcATTTCTATtTTTTaCTGTA		intergenic
8	chr13:85093766	CCTGtTTTgTtTGTTTTTCTGTA	LINC00375	intron 1
9	chr13:87651127	TACAGAAAcACAgAGAAAcCAGG	MIR4500HG	intron 1
10	chr14:85567936	CCTGATTTtTATtTTTTTCTaTA	FLRT2	intron 1
11	chr14:86450777	CCAcATTTCTATGcTTTTCTGgA		intergenic
12	chr15:58023149	TAaAGAAAcAaATAGAAATCTGG	ALDH1A2	intron 1
13	chr17:31152822	CCTtATTTtTATGTTTTTCTGTt	NF1	intron 1
14	chr18:21640391	CCTGATTTtTtTtTTTTTCTGTA		intergenic
15	chr18:29804666	CCAtATTTCTATGaTTTTCTaTA		intergenic
16	chr19:12396150	TACAGAAAAACATgaAAATaTGG	ZNF799	intron 1
17	chr19:12435983	TACAGAAAAACATgaAAATaTGG	ZNF443	intron 1
18	chr19:22856339	CCTcATTTtTcTGTTTTTCTGTA	ZNF723	intron 3
19	chr22:25011310	CCTGATTTtTgTGTTTTTtTGTA	KIAA1671	intron 1
20	chr3:46015647	CCTGATTTgTATtTTTTTCTcTA		intergenic
21	chr3:63497864	CCAGATgaCTATGTgTTTCTGTA	SYNPR	intron 3
22	chr4:7322969	CCCGtTTTtTATcTTTTTCTGTA	SORCS2	intron 1
23	chr4:60606464	TcCAGAAAAACAaAGAAATaAGG		intergenic
24	chr4:89365361	TACAGAAAAgCATAaAAATtTGG		intergenic
25	chr4:139741179	aACAGAAAcACtTAGAAATCTGG	MAML3	intron 2
26	chr4:150998516	CCTGATTTtTtTGTTTTTtTGTA	LRBA	intron 2
27	chr4:165165446	CCAGATTTCTATaTaTTTCTGTA	TMEM192	intron 2
28	chr5:14486941	CCTGATTTCTgTGTTTTTCTacA	TRIO	intron 47
29	chr5:19701084	TACAGAAAAAgATAaAAATaTGG	CDH18	intron 5
30	chr5:88651871	CCTGATTTCcATaTaTTTCTGTA	LINC00461	intron 2
31	chr5:105296348	TACAtAAAAACATAGAtATgGGG	AC.099520.1	intron 1
32	chr5:141553893	gAaAGAAAAACAgAGAAATCAGG	DIAPH1	intron 18
33	chr6:33426433	CCTGATTTCTcTtaTTTTCTGTA	SYNGAP1	intron 3
34	chr6:92774734	CCCtAggTCTATGTTTTTCTGTA		intergenic
35	chr6:146080372	aACAcAAAAACAgAGAAATCAGG	GRM1	intron 2
36	chr7:67830992	TACAGAAAgACAaAGAAATtTGG		intergenic
37	chr7:72899909	CCAGATTTtTATGaTTTTCTcTA	POM121	intron 5
38	chr7:100115160	CCAGATTTtTATcTTTTTCTGTt	TAF6	intron 2
39	chr7:108272738	CCAGAaTTCTATtTTTTTCTtTA	NRCAM	intron 3
40	chr8:16397344	aAaAaAAAAACATAGAAATCTGG		intergenic
41	chr8:22686004	TtgAGAAAtACATAGAAATCTGG		intergenic
42	chr8:137341828	CCAtcTTTCTATcTTTTTCTGTA		intergenic
43	chr9:70398258	CCGGATcTCTgTGTTTaTCTGTA	KLF9	intron 1
44	chr9:103648104	aACAGAAAAAaAcAGAAATCTGG		intergenic
45	chr9:108702372	TcCAGAAAAACtTtGAAATCAGG	AL358779.1	intron 1
46	chrX:48079160	TAaAGAAAAACAgAGAAAaCAGG		intergenic
